# Effectiveness of interval photography cameras for a survey of pollinator communities: Comparison with direct observation

**DOI:** 10.1002/aps3.70023

**Published:** 2025-09-27

**Authors:** Tomohiro Watazu, Masayoshi K. Hiraiwa, Masahito Inoue, Hideo Mishima, Atushi Ushimaru, Tetsuro Hosaka

**Affiliations:** ^1^ Development Technology Course, Graduate School for International Development and Cooperation Hiroshima University 1‐5‐1 Kagamiyama, Higashi‐Hiroshima City Hiroshima 739‐8529 Japan; ^2^ Department of Environmental Management Faculty of Agriculture, Kindai University, 3327‐204 Nara 631‐8505 Nakamachi Japan; ^3^ The Shimane Nature Museum of Mt. Sanbe 1121‐8 Tane, Sanbe‐cho Ohda 694‐0003 Shimane Japan; ^4^ Graduate School of Human Development and Environment Kobe University 3‐11 Tsurukabuto, Nada Kobe 657‐8501 Japan; ^5^ Transdisciplinary Science and Engineering Program, Graduate School of Advanced Science and Engineering Hiroshima University 1‐5‐1 Kagamiyama Higashi‐Hiroshima 739‐8529 Japan

**Keywords:** digital camera, Diptera, entomophilic type, flower traits, Hymenoptera, wetland plant community

## Abstract

**Premise:**

Pollinator communities have been surveyed through direct observation, which is labor intensive and difficult for monitoring nocturnal pollinators. Interval photography surveys are increasingly used, although the resulting data from pollinator community surveys have rarely been validated.

**Methods:**

We surveyed a pollinator community using both interval photography and direct observation and compared the two methods for differences in identification accuracy, pollinator composition, and number of pollinator visits detected.

**Results:**

Compared to direct observation, the species‐level identification rate of pollinators using interval photography was significantly lower, although the order‐level identification remained comparable. The number of pollinator observations was highly correlated between interval photography and direct observation, regardless of flower shape or pollinator groups. A longer observation period using interval photography (10 h), despite fewer observable flowers per camera, could yield a comparable or even greater number of pollinator observations than a much shorter period of direct observation (15 min).

**Discussion:**

Although interval photography has limitations for species‐level identification, it could be a powerful tool for surveying pollinator communities at higher taxonomic levels (e.g., order), including nocturnal pollinators, with reduced labor requirements and without human presence censuses.

Community‐wide plant–pollinator interactions have traditionally been investigated based on direct observation (Herrera, 1988; Kato and Miura, 1996; Kato, 1996; Elberling and Olesen, 1999; Memmott, 1999; Nakano and Washitani, 2003; Kato and Kawakita, 2004; Hegland and Totland, 2005; Lázaro et al., 2008, 2015; Hiraiwa and Ushimaru, 2017). This method involves real‐time field observation, recording, and sometimes capturing pollinators on target flowers. It presents several challenges: (1) it is labor‐intensive, requiring long hours in the field; (2) during periods of frequent pollinator visitation, it can be difficult to accurately record pollinator species, taxonomic groups, or their interactions with plants; (3) attempting to capture pollinators may disturb the environment; and (4) the process relies heavily on the observer's skill and experience. These challenges are further amplified when studying nocturnal pollinators.

Several studies have investigated pollinators using interval photography (Yokota and Yahara, 2012; Suetsugu and Hayamizu, 2014; Katsuhara et al., 2017; Nagai et al., 2022; Anderson et al., 2023). These studies used compact digital cameras that were set to take photographs at intervals ranging from 5 to 120 seconds, rather than using motion detection. Interval photography has several advantages compared to direct observation, as it allows extended observation periods with lower labor requirements, enables nighttime surveys that are difficult with direct observation, and can minimize the impact on pollinators that are sensitive to human presence. These characteristics make it a useful tool for long‐term, continuous pollinator surveys—including nocturnal observations—without causing significant disturbance or requiring extensive human labor. In a study by Yokota and Yahara (2012), interval photography was used to identify some pollinators of lilies with taxonomic resolution ranging from species level to family or order level. Suetsugu and Hayamizu (2014) revealed nocturnal moth pollination in *Platanthera* Rich. (Orchidaceae) using interval photography with a flash. Furthermore, Nagai et al. (2022) revealed the frequency and identity of pollinators of *Nelumbo nucifera* Gaertn. (Nelumbonaceae) at different times of the day using interval photography. Thus, interval photography is becoming a popular tool for pollinator surveys.

Interval photography, however, may have several potential disadvantages compared with direct observation. First, pollinator identification accuracy might be reduced due to out‐of‐focus images or partial body views in photographs. Particularly small pollinators (e.g., small bees, dipterans, and coleopterans) may be difficult to identify at species level from photographs due to resolution constraints. Second, detection rates may vary depending on pollinator group because residence time on flowers has been reported to vary among the groups (Herrera, 1989; Negoro, 2002). The detectability of pollinators can also differ depending on floral traits (e.g., flower shape) (Gilpin et al., 2017). Finally, because the number of flowers that can be observed is limited by the number of cameras available, interval photography can yield fewer pollinator observations per unit of time, although this limitation could be compensated for by longer survey periods of interval photography. Thus, interval photography could have limitations regarding lower accuracy of pollinator identification, bias toward some pollinator groups and floral types, and fewer pollinator observations.

While interval photography has been increasingly used for pollinator surveys, the quality and quantity of the resulting data have rarely been validated in comparison to direct observation surveys. In order to understand the strengths and limitations of interval photography, this study aims to compare pollinator data (community composition, taxonomic richness, and number of pollinator observations) collected by interval photography against direct observation. Because we found that it is often difficult to identify pollinators at species level using interval photography (see the Results section), we mainly compared the consistency of the two methods at order level. We addressed the following research questions:

(1) Is the rate of pollinator identification by interval photography lower than that by direct observation?
(2) Is the order‐level pollinator composition consistent between direct observation and interval photography?
(3) Are there any biases in pollinator detection rates among pollinator groups and flower shapes when using interval photography compared to direct observation?
(4) Does interval photography detect a lower number of pollinator visits per unit of time compared to direct observation? Can longer‐period interval photography compensate for this limitation?

In this study, the number of pollinator observations refers to the number of captured pollinators (with some individuals counted visually) and the number of pollinators recorded in photographs for direct observation and interval photography, respectively.

## METHODS

The study site, study plots, direct observation method, and floral trait measurements have been described in detail by Watazu et al. (2024). Therefore, a brief overview is provided in the following section.

## Study site

The survey was conducted in Akana Wetland, situated in Iinan Town, Shimane Prefecture (35°00′48″–00′50″N, 132°42′10″–42′25″E, 440 m a.s.l.). The region is classified as a warm temperate region despite the amount of snowfall in winter, with the annual average of deepest snowfall over the past 10 years being approximately 80 cm (Japan Meteorological Agency, 2023). Watazu et al. (2024) reported the pollinator fauna of many plants in the Akana Wetland, including plants with diverse floral traits that bloom from spring to autumn, using direct observation, and these data were used as a comparative dataset for interval photography in this study. The vegetation surrounding the Akana Wetland consisted predominantly of a *Rhododendron reticulatum* D. Don–*Pinus densiflora* Siebold & Zucc. community, whereas *Pogonia japonica* Rchb.f. (Orchidaceae), *Menyanthes trifoliata* L. (Menyanthaceae), and *Alnus japonica* (Thunb.) Steud. (Betulaceae) were dominant in wetland vegetation.

## Direct observation survey

Direct observations of 35 target species were conducted between 08:00 and 18:00 hours from April to November 2019 by capturing pollinators. Depending on the blooming area of the species, 1–26 1 m × 3 m plots were established for each plant species, with 15‐min direct observations conducted at a total of 275 plots. All pollinators observed in each plot during each 15‐min observation period were captured principally by a single researcher (T.W.). Each plant species was surveyed at least once in both morning and afternoon. Insects touching the stigma or anthers were counted as pollinators. In cases where pollinator capture was difficult, such as when multiple individuals visited the flowers simultaneously, we visually identified and counted them as far as possible. Similar direct observations using small plots and approximately 15‐min surveys have been conducted to quantify pollinator communities (Kato and Miura, 1996; Kato and Kawakita, 2004; Hegland and Totland, 2005; Lázaro et al., 2008; Gong and Huang, 2011). Additional survey details are provided in Watazu et al. (2024). For comparison with interval photography, we used the number of pollinator observations per 15‐min interval for each plant species. The number of pollinator observations here referred to the mean number of pollinators across plots for each plant species.

## Interval photography survey

We set up digital cameras (WG‐70; Ricoh, Tokyo, Japan) for 35 target plant species between April and November 2020 for the pollinator survey using interval photography. The automatic photography was conducted by the same researcher (T.W.) at 2‐min intervals using a flash, with the camera manually focused at a distance of approximately 30–50 cm from the flowers of each focal plant (Appendix S1, see Supporting Information with this article). This camera could take up to 1000 continuous photographs for approximately 33 h (day and night) when set at 2‐min intervals. We adopted 2‐min interval photography in this study because, with the same number of photographs, 2‐min interval photography (over a 24‐h period) detected more pollinators than 30‐s interval photography (over a 7‐h period) (Katsuhara et al., 2017). The height of the camera tripod and the lens focal length were adjusted to ensure that the flower stigmas and anthers were visible in the photographs. We also included a ruler in the first photograph to measure the size of pollinators. While we used photographs taken between 8:00 and 18:00 hours (10 h) in this study, photographs were taken during nighttime as well. The interval photography methods used here are similar to those used in other recent studies (Yokota and Yahara, 2012; Suetsugu and Hayamizu, 2014). Because it took us about 15 min to set up the camera for interval photography, the time spent in the field was almost equal to that of our 15‐min direct observation.

Insects touching the stigma or anthers were counted as pollinators. Pollinators were counted only once if the same species was photographed consecutively, following Yokota and Yahara (2012), Tsujimura et al. (2015), and Nishimura and Takayama (2023). Insects with an actual size smaller than 2 mm were excluded because of identification difficulties, using a ruler included in the first photograph as a reference. The photographed pollinators were identified at a low taxonomic level (species, genus, family, and order) by referring to a list of pollinators obtained using direct observation. For comparison with direct observation, we used both the number of pollinator observations per 15 min (equivalent to direct observation time) and per 10 h (the duration of one interval photography session) for each plant species. The number of pollinator observations here referred to the mean number of pollinators across cameras for each plant species.

## Floral trait measurements

To compare the effect of flower shape on the number of pollinator observations between the two methods, we classified flower shape as open or tube‐shaped following Hegland and Totland (2005) and Watazu et al. (2024) (Appendix S2), as flower morphology could affect observation frequency (e.g., complex flower shapes might be more difficult to photograph). To quantify the differences in the number of observed flowers between the two methods, the number of flowers of each plant in a plot (for the direct observation survey) was compared to that in the first photograph (for the interval photography survey).

## Data analysis

To evaluate sampling completeness between methods, we constructed rarefaction curves and analyzed sampling coverage and taxonomic richness using 22 plant species (24 species when including Formicidae) that had ≥10 pollinator observations in both methods (see Appendix S3 for details of the data analyses).

To assess the differences in community composition of pollinators between survey methods, we classified the pollinators of all 35 plant species into orders (Diptera, Hymenoptera [excluding Formicidae], Formicidae, Lepidoptera, Coleoptera, Hemiptera, Orthoptera, and Odonata). Hereafter, “Hymenoptera” refers specifically to Hymenoptera excluding Formicidae. Because the latter five orders were less frequent, we consolidated the data into three categories: Diptera, Hymenoptera, and other orders (combining the rest including Formicidae), and compared their proportions between survey methods using Fisher's exact probability test.

To compare pollinator assemblages between survey methods for each plant species, we classified plant species into entomophilic types using hierarchical cluster analysis and Ward's method. To ensure there was sufficient data to determine entomophilic types (Watazu et al., 2024), we only focused on plant species from which we had more than 10 pollinator observations with both direct observation and interval photography. The number of plant species used for the analyses were 22 and 24 when including and excluding the Formicidae, respectively (see Appendix S3 for details). For species that were classified into different entomophilic types between methods based on the cluster analysis, we tested differences in pollinator composition (Diptera, Hymenoptera, and other orders) using Fisher's exact probability test with Bonferroni correction.

To examine the correlation between pollinator observations across the two survey methods for each plant species, we conducted a Pearson's correlation test, using the number of pollinator observations for each 15‐min interval per flower for each method. The numbers of pollinator observations were log‐transformed after adding 0.0001 to improve the normality of data distribution (Yamamura, 1999) (Appendix S4).

We used generalized linear models (GLMs) with a Gaussian error distribution to investigate whether the effects of floral traits (open or tube‐shaped) and the pollinator group (Diptera or Hymenoptera) on the number of pollinator observations differed between survey methods (Appendix S3). To compare the differences in the number of pollinator observations between the two methods, we examined two separate GLMs with different observation time periods: (A) direct observation (15 min) vs. interval photography (15 min), and (B) direct observation (15 min) vs. interval photography (10 h). Model A compared the number of observations between the two methods within the same observation time period. Model B compared the number of observations between the two methods with actual observation time periods for each method, which is often much longer for interval photography than for direct observation (e.g., Katsuhara et al., 2017).

All statistical analyses were performed using R version 4.3.2 (R Core Team, 2023). We also referred to YList (http://ylist.info/) for plant species names and the Global Biodiversity Information Facility (GBIF Secretariat, 2023) and Schoch et al. (2020) for pollinator species names.

## RESULTS

### Pollinator community

**Figure 1:**
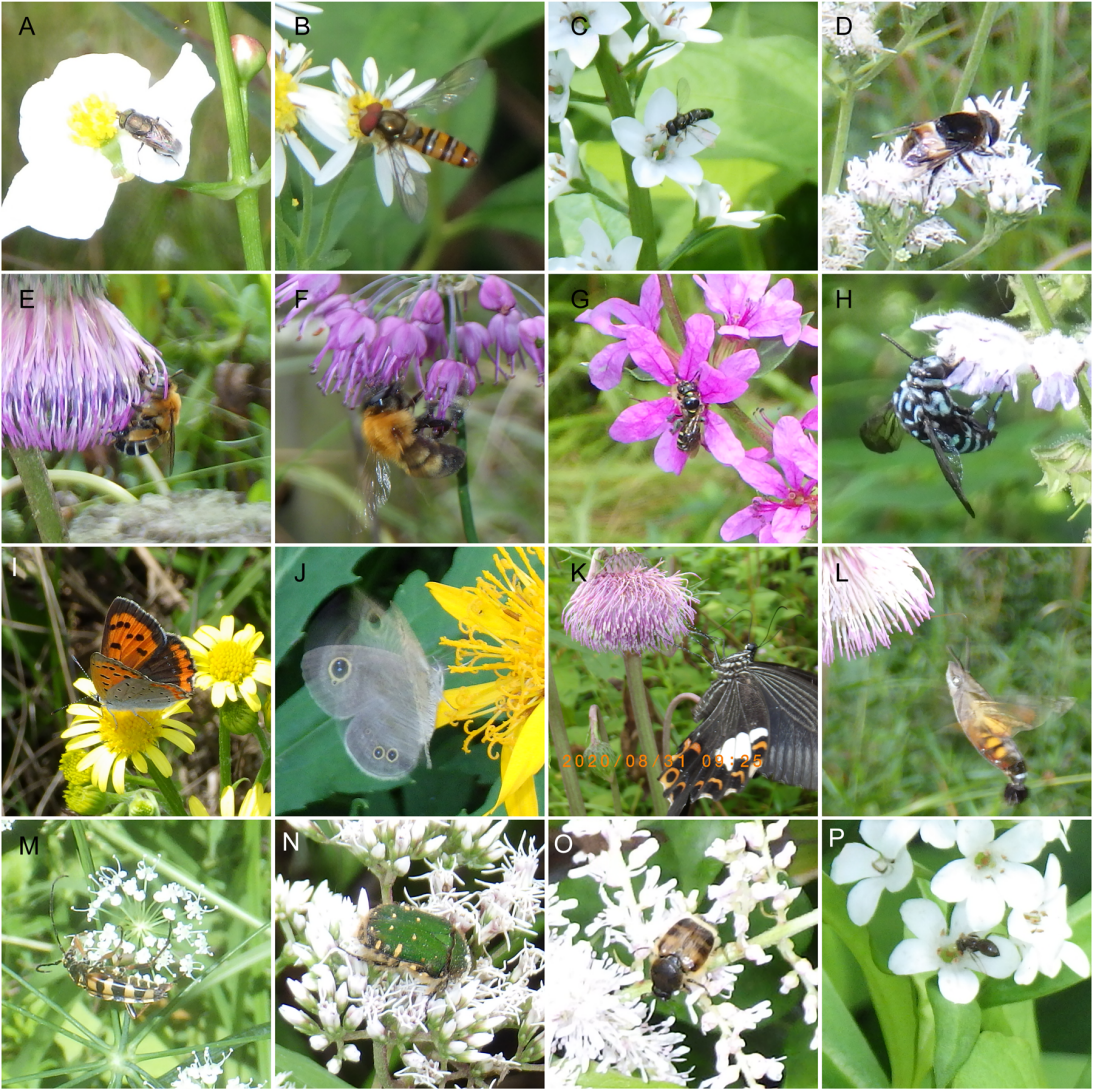
Examples of pollinators photographed using interval photography. (A) *Stomorhina obsoleta* visiting *Sagittaria aginashi* Makino; (B) *Episyrphus balteatus* visiting *Aster yomena* (Kitam.) Hondo; (C) *Paragus haemorrhous* visiting *Lysimachia clethroides* Duby; (D) *Phytomia zonata* visiting *Eupatorium lindleyanum* DC.; (E) *Amegilla florea* visiting *Cirsium sieboldii* Miq.; (F) *Bombus diversus* visiting *Allium thunbergii* G. Don; (G) *Ceratina* sp. visiting *Lythrum anceps* (Koehne) Makino; (H) *Thyreus decorus* visiting *Salvia japonica* Thunb.; (I) *Lycaena phlaeas* visiting *Senecio pierotii* Miq.; (J) *Ypthima argus* visiting *Ligularia japonica* (Thunb.) Less.; (K) *Papilio helenus* visiting *Cirsium sieboldii*; (L) *Macroglossum pyrrhosticta* visiting *Cirsium sieboldii*; (M) *Leptura ochraceofasciata* visiting *Cicuta virosa* L.; (N) *Gametis jucunda* visiting *Eupatorium lindleyanum*; (O) *Lasiotrichius succinctus* visiting *Astilbe microphylla* Knoll; (P) Formicidae sp. visiting *Lysimachia fortunei*.

We conducted 15‐min direct observations for a total of 68.8 h and counted 783 pollinators (1105 pollinators when including Formicidae), of which 754 were captured (791 when including Formicidae) (Appendix S2). For the interval photography survey, we set up a total of 170 cameras and took a total of 73,160 photographs. Of these, we excluded photographs in which the flower petals were closed or not clearly visible due to wind, resulting in a total of 62,428 photos (representing a total photography time of 2081 h). From those, we recorded 1357 pollinators (1870 pollinators when including Formicidae) (Figure 1, Appendix S2). Because small Formicidae (<5 mm in body length) were sometimes super‐abundant (>200 individuals) on flowers, which could mask the presence of other pollinators, we present results including Formicidae in the Supporting Information (Appendices S5–S10).

**Figure 2:**
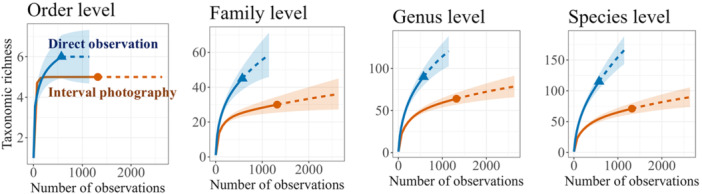
Rarefaction (solid lines) and extrapolation (dashed lines) curves of pollinator taxonomic richness, excluding Formicidae, based on the number of observations. Blue lines represent direct observation data and orange lines represent interval photography data, showing diversity patterns at different taxonomic levels. The shaded areas around each curve represent the 95% confidence intervals.

Based on interval photography, we identified 33.7% of the pollinators at the species level, 56.5% at the genus level, and 63.4% at the family level (Appendix S11). In direct observation, we identified 87.5%, 90.3%, and 99.9% of the pollinators at the species, genus, and family levels, respectively (Appendix S11). We could identify 32 and 13 dipteran, 34 and five hymenopteran, 25 and 24 lepidopteran, and eight and 10 coleopteran species using direct observation and interval photography, respectively (Appendices S12, S13). Rarefaction analysis showed that sampling coverage exceeded 90% at all taxonomic levels in both direct observation and interval photography, indicating sufficient coverage by each method (Figure 2, Appendix S14A). The I/D values of taxonomic richness (taxonomic richness of interval photography divided by that of direct observation) were 86% at order level, 81% at family level, 68% at genus level, and 48% at species level (Figure 2, Appendix S14A). Taxonomic richness for each plant species was significantly higher based on direct observation than interval photography at both the family and species levels (Appendices S15A, S15B, S16A).

**Figure 3:**
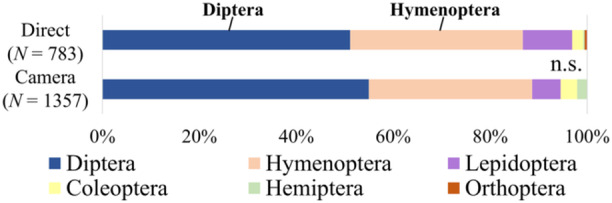
Functional group proportions of pollinators, excluding Formicidae, based on direct observation and interval photography in pollinator communities. The two proportions were not significantly different using Fisher's exact probability test.

Diptera were dominant in both direct observations (51.1% of the total pollinator observations) and interval photography (55.0% of the total pollinator observations) (Figure 3). The proportions of major pollinator categories (i.e., Diptera, Hymenoptera, and others) were not significantly different between the two methods at the community level (Fisher's exact probability test, *P* = 0.17).

**Figure 4:**
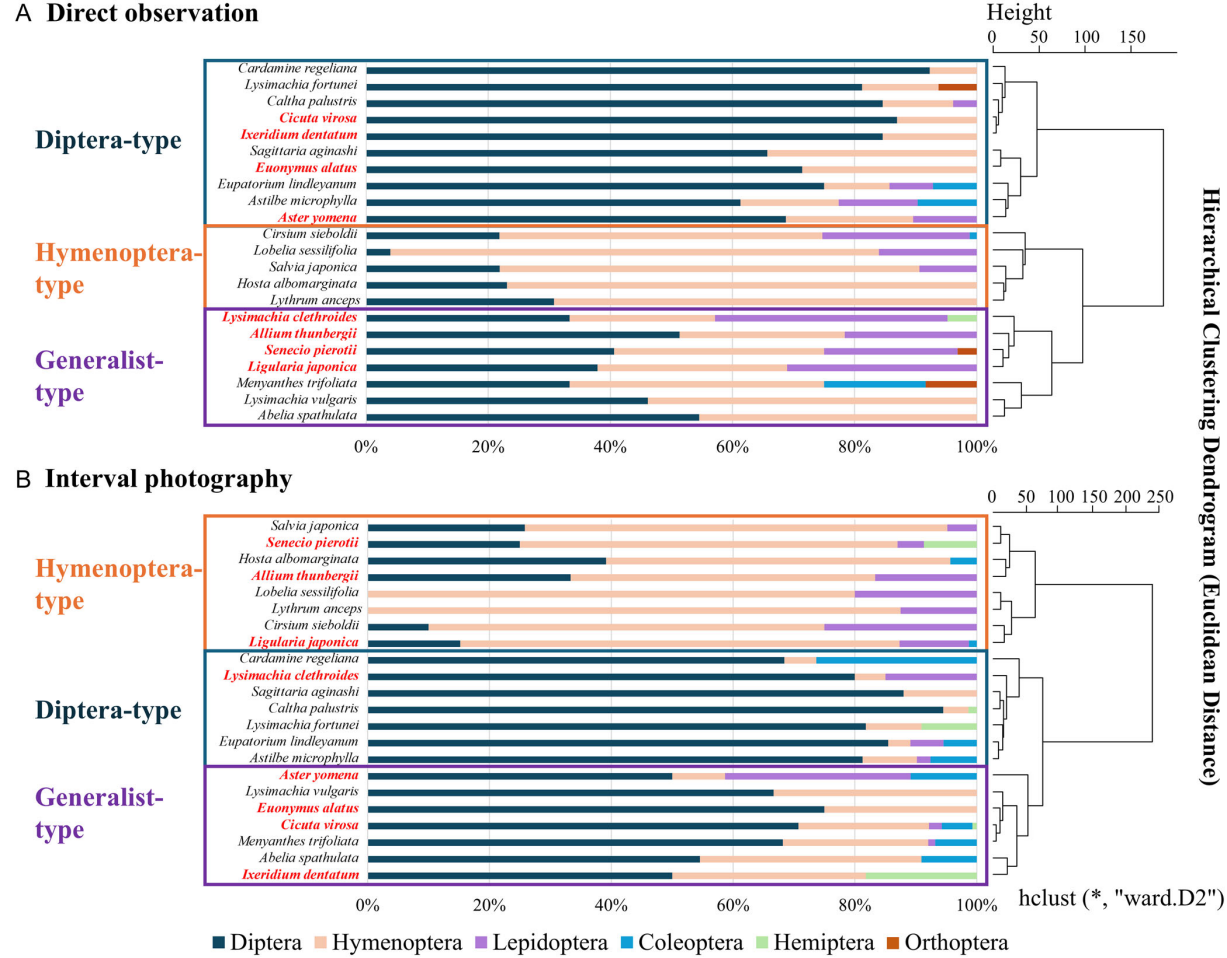
The percentage of the pollinator fauna, excluding Formicidae, based on direct observation (A) and interval photography (B). Each plant was classified according to hierarchical cluster analysis using Ward's method into Diptera‐type, Hymenoptera‐type, and generalist‐type based on the height of 100 in the dendrogram. Plant species that showed different pollinator types between methods are indicated in red.

The entomophilic type of each plant species was consistent between the two methods for 14 of the 22 species (Figure 4). Eight plant species were classified as different entomophilic types, but two of these eight species differed significantly for pollinator composition (Fisher's exact probability test with Bonferroni correction; Appendix S17). Plant species classified as different entomophilic types between the two survey methods had relatively diverse pollinator assemblages and were classified as G‐type (generalist‐type) based on both direct observation and interval photography.

### Flower traits and pollinator observations

**Figure 5:**
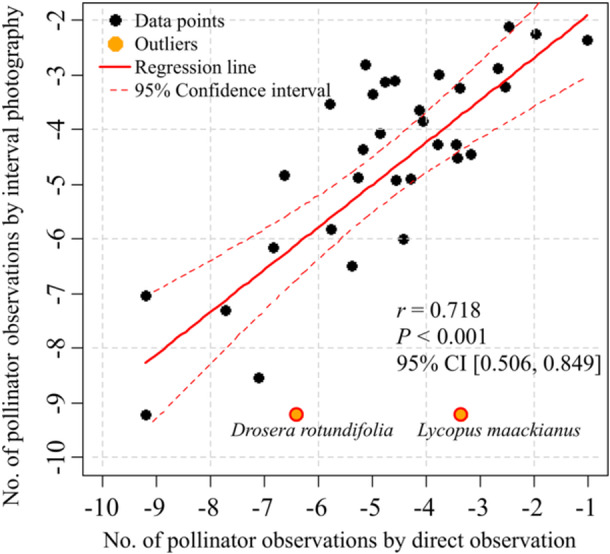
Graph showing the relationship between the number of pollinator observations, excluding Formicidae, based on interval photography and direct observation. Both variables were natural log‐transformed using the base *e* after adding 0.0001 to each value to avoid undefined logarithms for zero values. The number of pollinator observations was expressed as observations per 15‐min interval per flower. Pearson's correlation test was conducted to determine the correlation coefficient, *P*‐value, and 95% confidence interval. Outliers identified using Cook's distance are highlighted in the graph.

The two survey methods demonstrated a significant positive relationship in the number of pollinator observations per flower for each 15‐min period (Pearson's correlation test, *r* = 0.72, *P* < 0.001) (Figure 5), suggesting that the number of pollinator observations for the two methods were highly correlated across plant species. On the other hand, *Drosera rotundifolia* L. (Droseraceae) and *Lycopus maackianus* Makino (Lamiaceae) were revealed as outliers, with fewer observations recorded by interval photography (Figure 5).

The number of pollinator observations per flower was not significantly affected by flower shape (open or tube‐shaped) (GLM, *t* = −0.29, *P* = 0.77), and there was no significant interaction between the two survey methods and flower shape (GLM, *t* = −0.84, *P* = 0.40) (Table 1). This suggests that the number of pollinator observations between interval photography and direct observation did not differ between tube‐shaped and open flowers. Similarly, the number of pollinator observations per flower did not differ significantly between pollinator groups (Hymenoptera or Diptera) (GLM, *t* = 0.94, *P* = 0.35), and there was no significant interaction between observation method and pollinator group (GLM, *t* = −0.07, *P* = 0.94) (Table 1). This suggests that the number of observations of Hymenoptera and Diptera did not differ between interval photography and direct observation.

**Table 1:** Relationship between the number of pollinator observations (per 15‐min interval per flower) in direct observation and interval photography, method (direct observation and interval photography, direct observation was the baseline), flower shape (tube‐shaped or open, open was the baseline), and pollinator groups (Hymenoptera or Diptera, Hymenoptera was the baseline) analyzed using the generalized linear model. The number of pollinator observations, serving as the response variable, is categorized by method (direct observation or interval photography) and pollinator group (Hymenoptera or Diptera).

Response variable^a^	Explanatory variable	Estimate	SE	*t* value	*P*‐value
Log (no. of Hymenoptera and Diptera pollinator observations in direct observation and interval photography)	Intercept	−6.01	0.46	−13.17	<0.001
	Method (interval photography)	0.67	0.65	1.05	0.30
	Flower shape (tube)	−0.16	0.53	−0.29	0.77
	Pollinator group (Diptera)	0.50	0.53	0.94	0.35
	Method (interval photography) × Flower shape (tube)	−0.63	0.75	−0.84	0.40
	Method (interval photography) × Pollinator group (Diptera)	−0.05	0.75	−0.07	0.94

^a^ The response variable was log‐transformed after adding 0.0001 to each value to avoid undefined logarithms for zero values. The analysis includes interaction terms between the explanatory variables of method and flower traits, as well as method and pollinator groups.

Because a higher number of flowers were observed per survey for direct observation compared to interval photography, the number of pollinator observations per 15‐min survey was higher for direct observation than interval photography (GLM, *t* = −10.41, *P* < 0.001) (Table 2, Model A). However, when the number of flowers observed was considered, there was no significant difference between the two methods in the number of pollinator observations per flower in the same survey period (GLM, *t* = −0.05, *P* = 0.96) (Appendix S18A, Model A). These results indicate that a greater number of flowers can be observed in direct observation surveys compared to interval photography. Additionally, the number of pollinator observations by 10‐h interval photography was marginally insignificantly greater than that by 15‐min direct observation (GLM, *t* = 1.78, *P* = 0.08) (Table 2, Model B).

**Table 2:** Differences in the number of pollinator observations (excluding Formicidae) between direct observation and interval photography, based on generalized linear models. Two comparisons were performed: (A) between methods standardized to the same effort (15 min of direct observation vs. 15 min of interval photography), and (B) between 15 min of direct observation and 10 h of interval photography, reflecting typical operational durations in field studies. In both models, direct observation was used as the baseline level for the categorical variable method.

Response variable^a^	Explanatory variable	Estimate	SE	*t* value	*P*‐value
A: Log (no. of pollinators in direct observation and interval photography [per 15 min for both])	Intercept	1.16	0.06	18.20	<0.001
	Method (interval photography)	−1.01	0.09	−11.25	<0.001
B: Log (no. of pollinators in direct observation [per 15 min] and interval photography [per 10 h])	Intercept	1.16	0.14	8.10	<0.001
	Method (interval photography)	0.36	0.20	1.78	0.08

^a^ Pollinator counts were log‐transformed after adding 1 to avoid undefined values for zeros.

On average, the number of pollinator observations by direct observation (2.855 per 15 min) was 17.5 times greater than that by interval photography (0.163 per 15 min). In other words, 4.38 h of monitoring using interval photography (2‐min intervals) would be needed to achieve an equivalent number of pollinator observations to a 15‐min period of direct observation.

## DISCUSSION

### Comparison of direct observation and interval photography surveys

Interval photography was useful for describing pollinator community and composition at the order level for each plant species. However, there was a substantial difference in identification rates at the family level between direct observation (99.9%) and interval photography (63.4%), and taxonomic richness was significantly higher with direct observation. These results suggest that direct observation is particularly suitable for studies evaluating species diversity and composition of pollinators at the family level or below (e.g., Moquet et al., 2018; Purvis et al., 2020; Pindar and Raine, 2023). On the other hand, because detailed pollinator identification is not always necessary for studies examining pollinator communities (e.g., Hegland and Totland, 2005; Lázaro et al., 2008; Reverté et al., 2016), interval photography could be applied to such studies discussing pollinator compositions at higher taxonomic levels (e.g., order). Furthermore, a reference list of pollinator species based on direct observation greatly helped identification of photographed pollinators in our study. For example, *Paragus haemorrhous* (Syrphidae), *Amegilla florea* (Apidae), and *Bombus diversus* (Apidae) were captured during direct observation without any similar species, and thus we could identify these species even when photographs were incomplete. Therefore, the percentage of successful identification in our interval photography survey could have been lower without such a reference. Interval photography combined with direct observations provided better data with more accurate species identification.

On the other hand, the number of pollinator observations using interval photography showed a strong correlation with direct observation (Figure 5) regardless of pollinator group and flower shape (Table 1), demonstrating that interval photography is effective for quantifying the frequency of pollinator visitations. However, for *D. rotundifolia* and *L. maackianus*, interval photography recorded fewer pollinator visitations. Both species produce small flowers that are difficult to capture in large numbers with a camera, suggesting a potential limitation of interval photography for this flower type.

As we expected, interval photography detected considerably fewer pollinator visitations per unit of time than direct observation due to the limited number of focal flowers. However, a longer survey period of interval photography (10 h) could yield a comparable or even greater number of pollinator visitations than short periods of direct observation (15 min). In fact, the time period per survey is generally much longer for interval photography (7–15 h) (Yokota and Yahara, 2012; Suetsugu and Hayamizu, 2014; Nagai et al., 2022) than direct observation (10–20 min) (Kato and Miura, 1996; Hegland and Totland, 2005; Lázaro et al., 2008, 2015; but 1–2 h per survey in Weber and Kolb, 2013; Dzekashu et al., 2023). Although we used single battery and SD card in this study, the time period of interval photography can be further increased by replacing the battery and SD card, which requires less additional effort than direct observation.

Less required manpower is also an advantage of interval photography. Because it took about 15 min for one researcher to set up the camera for interval photography on site, the time spent in the field was almost equal to that of 15 min of direct observation by one researcher. Processing 10 h of interval photography data (300 photographs) by one researcher took approximately 5–20 min depending on the number of pollinator observations. Because direct observation also requires time for handling the specimens, the processing time for one researcher would be similar between methods. Therefore, we conclude that there is no substantial difference in time cost between the two methods.

In addition to the advantages detailed above, camera‐based methods can detect mammalian pollinators that are wary of human presence and traps (Kühn et al., 2017; Krauss et al., 2018). Interval photography could also be a powerful tool for studying nocturnal pollinators, given that direct observation is limited by visibility and increased physical and mental strain during nighttime fieldwork. Using interval photography may facilitate the discovery of previously unknown nocturnal pollinator communities and enable quantitative comparisons between diurnal and nocturnal pollinators.

Although we checked photographs manually in the present study, Bjerge et al. (2023) and Stark et al. (2023) used a machine learning approach to detect and identify pollinators in photographs. Combining interval photography with a highly accurate machine learning–based method to identify pollinators would allow the processing of large datasets, making interval photography a valuable tool for the long‐term monitoring of pollinator communities under climate change and human disturbance.

### Limitations of this study

In the present study, the direct observation and interval photography surveys were conducted over different years. Therefore, the observed differences in pollinator composition in the same plant species between the two methods might be partly due to population fluctuations in pollinators from year to year. This study was a part of a larger project to identify wetland pollinator communities, and the scope of the original study project did not include comparing direct observation and interval photography survey data. Although it would have been preferable if the study had been specifically designed with this in mind, we think that our dataset covering pollinator communities of 35 wetland plants with both direct observation and interval photography is still useful to evaluate similarities and differences between these survey methods. The high consistency in detected pollinator community metrics between methods (e.g., Figures 3, 4, 5) indicates that pollinator communities were not largely changed between the two years except for those recorded at some generalist plants (Appendix S17).

Our study did not control environmental variables (e.g., wind, light, temperature) or possible influence of the camera flash on pollinator behavior (e.g., shortening the residence time), which may affect image quality and data reliability of interval photography. The influence of these factors should be assessed in future studies. Moreover, this study was limited to one wetland plant community, and further investigation is necessary in grassland and forest communities, which are known to be dominated by Hymenoptera, unlike wetlands (Shinjo et al., 2014). Many bees could not be identified at the species level using interval photography in the present study. Therefore, the utility of this method in bee‐dominated communities requires further investigation.

### Conclusions

Our research highlights the strengths and limitations of interval photography compared to direct observation. Interval photography demonstrated comparable results with direct observation in identifying pollinators at the order level, and there was no difference in the number of pollinator observations between the two methods, regardless of flower morphology or pollinator groups. Furthermore, although fewer flowers could be observed with a camera compared to direct observation, longer observation times of interval photography can yield a comparable number of pollinator observations with less effort. These results indicate that interval photography can be applied to pollinator studies that do not require taxonomic precision beyond the order level (Hegland and Totland, 2005; Lázaro et al., 2008; Gong and Huang, 2011; Watazu et al., 2024). Interval photography can be particularly useful for studying nocturnal pollinators. While pollinator community observations have traditionally been limited to daytime (Macgregor and Scott‐Brown, 2020), nocturnal pollinators have been documented in many plant species (Suetsugu and Hayamizu, 2014; Reith and Zoba, 2016; Funamoto and Sugiura, 2016, 2020; Funamoto and Ohashi, 2017; Katsuhara et al., 2017; Macgregor and Scott‐Brown, 2020). Additionally, interval photography has an advantage as it is non‐invasive for insects and has minimal environmental impact, making it suitable for long‐term monitoring of pollinator communities. Although direct observation remains important for studies requiring higher levels of taxonomic precision, interval photography could be a powerful complement for obtaining comprehensive and long‐term datasets on pollinators.

## AUTHOR CONTRIBUTIONS

T.W., A.U., and T.H. designed the study. T.W. performed data collection, and T.W., M.K.H., M.I., and H.M. classified the plant and insect species. W.T. drafted the manuscript, and M.K.H., A.U., and T.H. performed the analyses and interpreted the data. All authors revised the draft and approved the final version of the manuscript.

## Supporting information


**Appendix S1.** Setup of interval photography for pollinator observation. The digital camera was mounted on a tripod approximately 30–50 cm from the target flowers and set to take photographs with flash at 2‐min intervals.
**Appendix S2.** Flower traits and number of pollinators in each plant species using direct observation and interval photography. (A) represents data excluding Formicidae, (B) represents data including Formicidae.
**Appendix S3.** Details for rarefaction and generalized linear model analyses.
**Appendix S4.** Residual diagnostics of GLM analysis with (A) non‐transformed and (B) log‐transformed response variables. These figures show that log‐transformed data improved homogeneity of variances (residuals vs. fitted plots) and normality of data distribution (normal Q‐Q plots).
**Appendix S5.** Rarefaction (solid lines) and extrapolation (dashed lines) curves of pollinator taxonomic richness, including Formicidae, based on the number of observations.
**Appendix S6.** Functional group proportions of pollinators, including Formicidae, for direct observation and interval photography in pollinator communities.
**Appendix S7.** The percentage of the pollinator fauna, including Formicidae, of interval photography and direct observation.
**Appendix S8.** Graph showing the relationship between the number of pollinator observations, including Formicidae, in interval photography and direct observation.
**Appendix S9.** Differences in the number of pollinator observations (including Formicidae) between direct observation and interval photography, based on generalized linear models.
**Appendix S10.** Differences in the number of pollinator observations per flower (including Formicidae) between direct observation and interval photography, based on generalized linear models.
**Appendix S11.** Classifiable proportions of pollinators in direct and interval photography.
**Appendix S12.** The number of pollinator observations for each taxon in captured pollinators during direct observation and photographed pollinators using interval photography.
**Appendix S13.** The number of pollinator species in each order identified by direct observation and interval photography.
**Appendix S14.** Comparison of sampling coverage and taxonomic richness between direct observation and interval photography methods across taxonomic hierarchies.
**Appendix S15.** Observation‐based rarefaction (solid lines) and extrapolation (dashed lines) curves showing pollinator diversity at different taxonomic levels for each plant species.
**Appendix S16.** Comparison of sampling coverage and taxonomic richness between direct observation and interval photography methods for each plant species across taxonomic hierarchies.
**Appendix S17.** Plant species with different entomophilic types as recorded using direct observation and interval photography, and statistical significance in the proportion of pollinator groups based on Fisher's exact test with Bonferroni correction.
**Appendix S18.** Differences in the number of pollinator observations per flower (excluding Formicidae) between direct observation and interval photography, based on generalized linear models.

## Data Availability

All data used in this study are provided within the article and Supporting Information.
