# Supplementary material for: Effectiveness of interval photography cameras for a survey of pollinator communities: Comparison with direct observation
Source: Appl Plant Sci. 2025 Sep 27;13(5):e70023. doi: 10.1002/aps3.70023 (PMC12542806; doi:10.1002/aps3.70023)
Supplement: Supplementary file 1 — Appendix S1. Setup of interval photography for pollinator observation. The digital camera was mounted on a tripod approximately 30–50 cm from the target flowers and set to take photographs with flash at 2‐min intervals. Appendix S2. Flower traits and number of pollinators in each plant species using direct observation and interval photography. (A) represents data excluding Formicidae, (B) represents data including Formicidae. Appendix S3. Details for rarefaction and generalized linear model analyses. Appendix S4. Residual diagnostics of GLM analysis with (A) non‐transformed and (B) log‐transformed response variables. These figures show that log‐transformed data improved homogeneity of variances (residuals vs. fitted plots) and normality of data distribution (normal Q‐Q plots). Appendix S5. Rarefaction (solid lines) and extrapolation (dashed lines) curves of pollinator taxonomic richness, including Formicidae, based on the number of observations. Appendix S6. Functional group proportions of pollinators, including Formicidae, for direct observation and interval photography in pollinator communities. Appendix S7. The percentage of the pollinator fauna, including Formicidae, of interval photography and direct observation. Appendix S8. Graph showing the relationship between the number of pollinator observations, including Formicidae, in interval photography and direct observation. Appendix S9. Differences in the number of pollinator observations (including Formicidae) between direct observation and interval photography, based on generalized linear models. Appendix S10. Differences in the number of pollinator observations per flower (including Formicidae) between direct observation and interval photography, based on generalized linear models. Appendix S11. Classifiable proportions of pollinators in direct and interval photography. Appendix S12. The number of pollinator observations for each taxon in captured pollinators during direct observation and photographed pollinato [file APS3-13-e70023-s001.docx]

**Supporting information for “Effectiveness of interval photography cameras for a survey of pollinator communities: Comparison with direct observation”**

**Appendix S1.** Setup of interval photography for pollinator observation. The digital camera was mounted on a tripod approximately 30–50 cm from the target flowers and set to take photographs with flash at 2-min intervals.


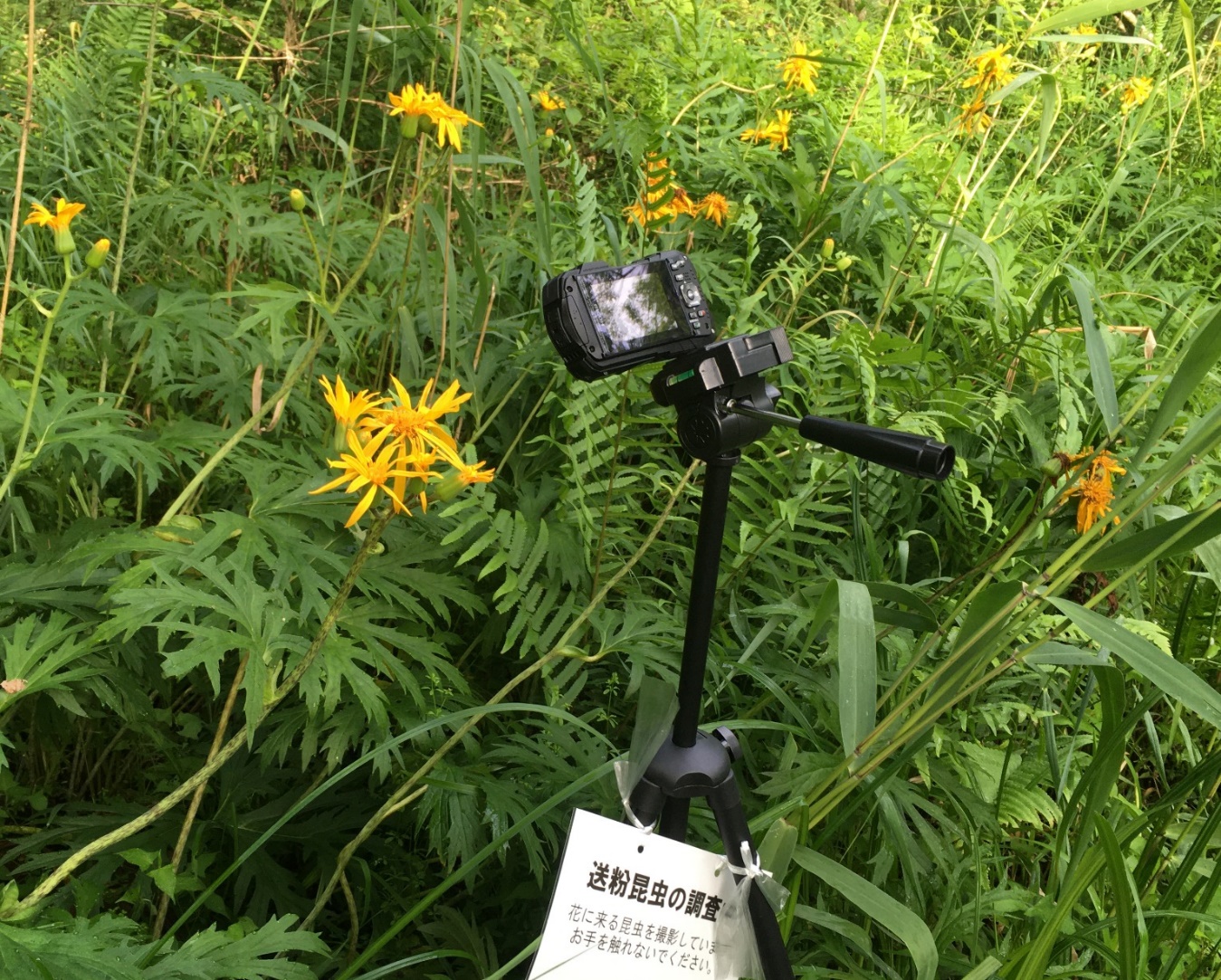


**Appendix S2.** Flower traits and number of pollinators in each plant species using direct observation and interval photography. (A) represents data excluding Formicidae, (B) represents data including Formicidae.

1. Excluding Formicidae

| Flower species | Flower shape | Direct observation | | | | | |  | Interval photography | | | | | |
| --- | --- | --- | --- | --- | --- | --- | --- | --- | --- | --- | --- | --- | --- | --- |
|  |  | No of survey | Survey time (min) | No of flowers per plot | No of pollinators counted | No of pollinators per 15 min | No of pollinators per 10 h |  | No of cameras | No of photos taken | No of flowers per photo | No of pollinators taken | No of pollinators per 15 min | No of pollinators per 10 h |
| *Abelia spathulata* | Tube | 4 | 60 | 83 | 11 | 2.75 | 110 |  | 3 | 1548 | 1 | 11 | 0.05 | 2.13 |
| *Allium thunbergii* | Tube | 12 | 180 | 410 | 37 | 3.08 | 123.3 |  | 4 | 2058 | 10 | 48 | 0.17 | 7 |
| *Aster glehnii* | Open | 9 | 135 | 923 | 37 | 4.11 | 164.4 |  | 4 | 1219 | 21 | 5 | 0.03 | 1.23 |
| *Aster yomena* | Open | 12 | 180 | 178 | 48 | 4 | 160 |  | 4 | 1285 | 5 | 46 | 0.27 | 10.74 |
| *Astilbe microphylla* | Open | 7 | 105 | 3672 | 31 | 4.57 | 177.1 |  | 4 | 1876 | 112 | 224 | 0.9 | 35.82 |
| *Caltha palustris* | Open | 19 | 285 | 236 | 26 | 1.37 | 54.7 |  | 4 | 1231 | 7 | 73 | 0.44 | 17.79 |
| *Cardamine regeliana* | Open | 11 | 165 | 404 | 13 | 1.18 | 47.3 |  | 3 | 900 | 5 | 19 | 0.16 | 6.33 |
| *Cicuta virosa* | Open | 5 | 75 | 4821 | 23 | 4.6 | 184 |  | 4 | 1973 | 262 | 140 | 0.53 | 21.29 |
| *Cirsium sieboldii* | Tube | 32 | 480 | 20 | 87 | 2.72 | 108.8 |  | 4 | 1858 | 2 | 60 | 0.24 | 9.69 |
| *Drosera rotundifolia* | Open | 9 | 135 | 49 | 15 | 1.67 | 66.7 |  | 8 | 792 | 1 | 0 | 0 | 0 |
| *Eriocaulon sikokianum* | Open | 8 | 120 | 550 | 13 | 1.63 | 65 |  | 4 | 1364 | 8 | 4 | 0.02 | 0.88 |
| *Euonymus alatus* | Open | 4 | 60 | 527 | 14 | 3.5 | 140 |  | 3 | 1473 | 2 | 16 | 0.08 | 3.26 |
| *Eupatorium lindleyanum* | Tube | 9 | 135 | 9076 | 28 | 3.11 | 124.4 |  | 4 | 1864 | 384 | 55 | 0.22 | 8.85 |
| *Hosta albomarginata* | Tube | 6 | 90 | 28 | 13 | 2.17 | 86.7 |  | 4 | 1363 | 3 | 23 | 0.13 | 5.06 |
| *Impatiens textorii* | Tube | 8 | 120 | 148 | 20 | 2.5 | 100 |  | 5 | 1458 | 2 | 8 | 0.04 | 1.65 |
| *Isachne globosa* | Open | 7 | 105 | 2851 | 14 | 2 | 80 |  | 4 | 1476 | 53 | 1 | 0.01 | 0.2 |
| *Ixeridium dentatum* | Open | 4 | 60 | 329 | 13 | 3.25 | 130 |  | 5 | 1351 | 3 | 22 | 0.12 | 4.89 |
| *Ligularia japonica* | Open | 9 | 135 | 39 | 29 | 3.22 | 128.9 |  | 5 | 2701 | 2 | 79 | 0.22 | 8.77 |
| *Lobelia sessilifolia* | Tube | 11 | 165 | 72 | 25 | 2.27 | 90.9 |  | 4 | 1473 | 4 | 10 | 0.05 | 2.04 |
| *Lycopus maackianus* | Tube | 11 | 165 | 766 | 13 | 1.18 | 47.3 |  | 4 | 1589 | 13 | 0 | 0 | 0 |
| *Lysimachia clethroides* | Open | 4 | 60 | 164 | 21 | 5.25 | 210 |  | 5 | 1500 | 9 | 20 | 0.1 | 4 |
| *Lysimachia fortunei* | Open | 7 | 105 | 103 | 16 | 2.43 | 91.4 |  | 3 | 1485 | 8 | 22 | 0.11 | 4.44 |
| *Lysimachia vulgaris* | Open | 5 | 75 | 470 | 13 | 2.6 | 104 |  | 5 | 1418 | 11 | 27 | 0.14 | 5.71 |
| *Lythrum anceps* | Tube | 9 | 135 | 123 | 13 | 1.44 | 57.8 |  | 4 | 2102 | 24 | 16 | 0.06 | 2.28 |
| *Menyanthes trifoliata* | Tube | 11 | 165 | 132 | 12 | 1.09 | 43.6 |  | 4 | 1789 | 8 | 88 | 0.37 | 14.76 |
| *Mosla dianthera* | Tube | 5 | 75 | 506 | 26 | 5.2 | 208 |  | 6 | 1029 | 2 | 2 | 0.01 | 0.58 |
| *Persicaria thunbergii* | Open | 20 | 300 | 513 | 51 | 2.55 | 102 |  | 3 | 962 | 8 | 8 | 0.06 | 2.49 |
| *Platanthera nipponica* | Tube | 7 | 105 | 25 | 0 | 0 | 0 |  | 24 | 9214 | 3 | 3 | 0.002 | 0.1 |
| *Pogonia japonica* | Tube | 7 | 105 | 10 | 3 | 0.43 | 17.1 |  | 6 | 3176 | 1 | 5 | 0.01 | 0.47 |
| *Sagittaria aginashi* | Open | 9 | 135 | 11 | 35 | 3.89 | 155.6 |  | 5 | 2194 | 2 | 50 | 0.17 | 6.84 |
| *Salvia japonica* | Tube | 13 | 195 | 156 | 32 | 2.46 | 98.5 |  | 4 | 1569 | 11 | 62 | 0.3 | 11.85 |
| *Senecio pierotii* | Open | 6 | 90 | 78 | 32 | 5.33 | 213.3 |  | 4 | 1301 | 21 | 208 | 1.2 | 47.96 |
| *Utricularia bifida* | Tube | 4 | 60 | 22 | 0 | 0 | 0 |  | 5 | 1179 | 1 | 0 | 0 | 0 |
| *Utricularia uliginosa* | Tube | 4 | 60 | 33 | 0 | 0 | 0 |  | 4 | 937 | 2 | 0 | 0 | 0 |
| *Viola verecunda* | Tube | 13 | 195 | 110 | 19 | 1.46 | 58.5 |  | 4 | 1721 | 1 | 2 | 0.01 | 0.35 |

1. Including Formicidae

| Flower species | Flower shape | Direct observation | | | | | |  | Interval photography | | | | | |
| --- | --- | --- | --- | --- | --- | --- | --- | --- | --- | --- | --- | --- | --- | --- |
|  |  | No of survey | Survey time (min) | No of flowers per plot | No of pollinators counted | No of pollinators per 15 min | No of pollinators per 10 h |  | No of cameras | No of photos taken | No of flowers per photo | No of pollinators taken | No of pollinators per 15 min | No of pollinators per 10 h |
| *Abelia spathulata* | Tube | 4 | 60 | 83 | 12 | 3.00 | 120.0 |  | 3 | 1548 | 1 | 11 | 0.05 | 2.1 |
| *Allium thunbergii* | Tube | 12 | 180 | 410 | 37 | 3.08 | 123.3 |  | 4 | 2058 | 10 | 48 | 0.17 | 7.0 |
| *Aster glehnii* | Open | 9 | 135 | 923 | 37 | 4.11 | 164.4 |  | 4 | 1219 | 21 | 42 | 0.26 | 10.3 |
| *Aster yomena* | Open | 12 | 180 | 178 | 48 | 4.00 | 160.0 |  | 4 | 1285 | 5 | 46 | 0.27 | 10.7 |
| *Astilbe microphylla* | Open | 7 | 105 | 3672 | 185 | 26.43 | 1057.1 |  | 4 | 1876 | 112 | 331 | 1.32 | 52.9 |
| *Caltha palustris* | Open | 19 | 285 | 236 | 44 | 2.32 | 92.6 |  | 4 | 1231 | 7 | 73 | 0.44 | 17.8 |
| *Cardamine regeliana* | Open | 11 | 165 | 404 | 13 | 1.18 | 47.3 |  | 3 | 900 | 5 | 20 | 0.17 | 6.7 |
| *Cicuta virosa* | Open | 5 | 75 | 4821 | 137 | 27.40 | 1096.0 |  | 4 | 1973 | 262 | 373 | 1.42 | 56.7 |
| *Cirsium sieboldii* | Tube | 32 | 480 | 20 | 87 | 2.72 | 108.8 |  | 4 | 1858 | 2 | 60 | 0.24 | 9.7 |
| *Drosera rotundifolia* | Open | 9 | 135 | 49 | 15 | 1.67 | 66.7 |  | 8 | 792 | 1 | 0 | 0 | 0 |
| *Eriocaulon sikokianum* | Open | 8 | 120 | 550 | 13 | 1.63 | 65.0 |  | 4 | 1364 | 8 | 4 | 0.02 | 0.9 |
| *Euonymus alatus* | Open | 4 | 60 | 527 | 29 | 7.25 | 290.0 |  | 3 | 1473 | 2 | 50 | 0.25 | 10.2 |
| *Eupatorium lindleyanum* | Tube | 9 | 135 | 9076 | 28 | 3.11 | 124.4 |  | 4 | 1864 | 384 | 55 | 0.22 | 8.9 |
| *Hosta albomarginata* | Tube | 6 | 90 | 28 | 13 | 2.17 | 86.7 |  | 4 | 1363 | 3 | 23 | 0.13 | 5.1 |
| *Impatiens textorii* | Tube | 8 | 120 | 148 | 20 | 2.50 | 100.0 |  | 5 | 1458 | 2 | 8 | 0.04 | 1.6 |
| *Isachne globosa* | Open | 7 | 105 | 2851 | 14 | 2.00 | 80.0 |  | 4 | 1476 | 53 | 1 | 0.01 | 0.2 |
| *Ixeridium dentatum* | Open | 4 | 60 | 329 | 13 | 3.25 | 130.0 |  | 5 | 1351 | 3 | 22 | 0.12 | 4.9 |
| *Ligularia japonica* | Open | 9 | 135 | 39 | 29 | 3.22 | 128.9 |  | 5 | 2701 | 2 | 91 | 0.25 | 10.1 |
| *Lobelia sessilifolia* | Tube | 11 | 165 | 72 | 25 | 2.27 | 90.9 |  | 4 | 1473 | 4 | 10 | 0.05 | 2.0 |
| *Lycopus maackianus* | Tube | 11 | 165 | 766 | 14 | 1.27 | 50.9 |  | 4 | 1589 | 13 | 17 | 0.08 | 3.2 |
| *Lysimachia clethroides* | Open | 4 | 60 | 164 | 30 | 7.50 | 300.0 |  | 5 | 1500 | 9 | 59 | 0.30 | 11.8 |
| *Lysimachia fortunei* | Open | 7 | 105 | 103 | 21 | 3.00 | 120.0 |  | 3 | 1485 | 8 | 39 | 0.20 | 7.9 |
| *Lysimachia vulgaris* | Open | 5 | 75 | 470 | 13 | 2.60 | 104.0 |  | 5 | 1418 | 11 | 27 | 0.14 | 5.7 |
| *Lythrum anceps* | Tube | 9 | 135 | 123 | 13 | 1.44 | 57.8 |  | 4 | 2102 | 24 | 16 | 0.06 | 2.3 |
| *Menyanthes trifoliata* | Tube | 11 | 165 | 132 | 16 | 1.45 | 58.2 |  | 4 | 1789 | 8 | 90 | 0.38 | 15.1 |
| *Mosla dianthera* | Tube | 5 | 75 | 506 | 26 | 5.20 | 208.0 |  | 6 | 1029 | 2 | 2 | 0.01 | 0.6 |
| *Persicaria thunbergii* | Open | 20 | 300 | 513 | 52 | 2.60 | 104.0 |  | 3 | 962 | 8 | 8 | 0.06 | 2.5 |
| *Platanthera nipponica* | Tube | 7 | 105 | 25 | 0 | 0 | 0 |  | 24 | 9214 | 3 | 3 | 0.002 | 0.1 |
| *Pogonia japonica* | Tube | 7 | 105 | 10 | 3 | 0.43 | 17.1 |  | 6 | 3176 | 1 | 5 | 0.01 | 0.5 |
| *Sagittaria aginashi* | Open | 9 | 135 | 11 | 35 | 3.89 | 155.6 |  | 5 | 2194 | 2 | 61 | 0.21 | 8.3 |
| *Salvia japonica* | Tube | 13 | 195 | 156 | 32 | 2.46 | 98.5 |  | 4 | 1569 | 11 | 62 | 0.30 | 11.9 |
| *Senecio pierotii* | Open | 6 | 90 | 78 | 32 | 5.33 | 213.3 |  | 4 | 1301 | 21 | 209 | 1.20 | 48.2 |
| *Utricularia bifida* | Tube | 4 | 60 | 22 | 0 | 0 | 0 |  | 5 | 1179 | 1 | 0 | 0 | 0 |
| *Utricularia uliginosa* | Tube | 4 | 60 | 33 | 0 | 0 | 0 |  | 4 | 937 | 2 | 0 | 0 | 0 |
| *Viola verecunda* | Tube | 13 | 195 | 110 | 19 | 1.46 | 58.5 |  | 4 | 1721 | 1 | 4 | 0.02 | 0.7 |

**Appendix S3.** Details for rarefaction and generalized linear model analyses.

**Rarefaction analysis**

We created rarefaction curves for both direct observation and interval photography based on the number of pollinator observations and taxonomic richness at different taxonomic resolutions (order, family, genus, and species levels). To show the proportion of taxonomic richness from interval photography relative to direct observation, we calculated I/D values (taxonomic richness of interval photography divided by taxonomic richness of direct observation). Similarly, we created rarefaction curves for each plant species. Differences in taxonomic richness between the two methods at each taxonomic level were tested using paired *t*-tests.

**Hierarchical cluster analysis for entomophilic types**

Although Formicidae are generally considered ineffective pollinators and nectar thieves (Beattie et al., 1984, 1985; Hull and Beattie, 1988; Dutton and Frederickson, 2012), they can function as effective pollinators for some plant species (Kuriakose et al., 2018; Natsume et al., 2022; Wyatt et al., 2023). Therefore, we conducted all analyses using both datasets (including and excluding Formicidae in Hymenoptera) separately. When excluding the Formicidae, we classified 22 plant species into three types—Diptera-type (D-type), Hymenoptera-type (H-type), and generalist-type (G-type)—based on pollinator order compositions (Diptera, Hymenoptera, Lepidoptera, Coleoptera, Hemiptera, and Orthoptera). When including Formicidae, we classified 24 plant species into four types by adding ant-type (A-type).

**GLM model for floral traits and pollinator groups**

Considering the distribution characteristics of the data, we assumed a Gaussian (normal) distribution for the generalized linear model (GLM). The response variable was log-transformed (log(N+0.0001)) to better meet the assumptions of normality and homoscedasticity. As clearly shown in the residual diagnostic results in Appendix S4, the log-transformed model demonstrated substantially improved normality in the Q-Q plot and homoscedasticity in the Residuals vs. Fitted plot compared to the non-transformed model. The response variables were the number of Hymenoptera and Diptera pollinator observations per 15-min interval per flower for each plant species, obtained from the two methods. The response variables were transformed into log (N+0.0001) before analyses to ensure normality. The explanatory variables included observation method (direct observation or interval photography), flower shape (open or tube), pollinator group (Hymenoptera or Diptera), and the two-way interactions between observation method and flower shape and between observation method and pollinator group. All explanatory variables were treated as categorical variables.

**GLM model for comparing pollinator observations between the two methods**

In all models (A and B), the explanatory variables were the number of flowers and observation method (direct observation or interval photography), with direct observation set as the baseline level. We used GLMs with a Gaussian error distribution. The response variables were the log-transformed number of pollinators per 15 min or per 10 h for each plant species, obtained from the two methods. Prior to log-transformation, a value of 1 was added to each observation to avoid undefined logarithms for zero values. Additionally, to compare the two methods while taking into account the number of observed flowers, we conducted similar analyses by changing only the response variable to the log-transformed number of pollinators per flower per 15 min or per 10 h for each plant species from both methods.

**REFERENCES**

Beattie, A. J., C. Turnbull, R. B. Knox, and E. G. Williams. 1984. Ant inhibition of pollen function: A possible reason why ant pollination is rare. *American Journal of Botany* 71: 421–426.

Beattie, A. J., C. Turnbull, T. Hough, S. Jobson, and R. B. Knox. 1985. The vulnerability of pollen and fungal spores to ant secretions: Evidence and some evolutionary implications. *American Journal of Botany* 72: 606–614.

Dutton, E. M., and M. E. Frederickson. 2012. Why ant pollination is rare: New evidence and implications of the antibiotic hypothesis. *Arthropod–Plant Interactions* 6: 561–569.

Hull, D. A., and A. J. Beattie. 1988. Adverse effects on pollen exposed to *Atta texana* and other North American ants: Implications for ant pollination. *Oecologia* 75: 153–155.

Kuriakose, G., P. A. Sinu, and K. R. Shivanna. 2018. Ant pollination of *Syzygium occidentale*, an endemic tree species of tropical rain forests of the Western Ghats, India. *Arthropod-Plant Interactions* 12: 647–655.

Natsume, K., S. Hayashi, and T. Miyashita. 2022. Ants are effective pollinators of common buckwheat *Fagopyrum esculentum*. *Agricultural and Forest Entomology* 24(3): 446–452.

Wyatt, R., B. D. Beliveau, and G. E. Wyatt. 2023. Reproductive biology of *Euonymus americanus* (Celastraceae): Pollination by ants and cockroaches. *Journal of the Torrey Botanical Society* 150(4): 538–548.

**Appendix S4.** Residual diagnostics of GLM analysis with (A) non-transformed and (B) log-transformed response variables. These figures show that log-transformed data improved homogeneity of variances (residuals vs. fitted plots) and normality of data distribution (normal Q-Q plots).


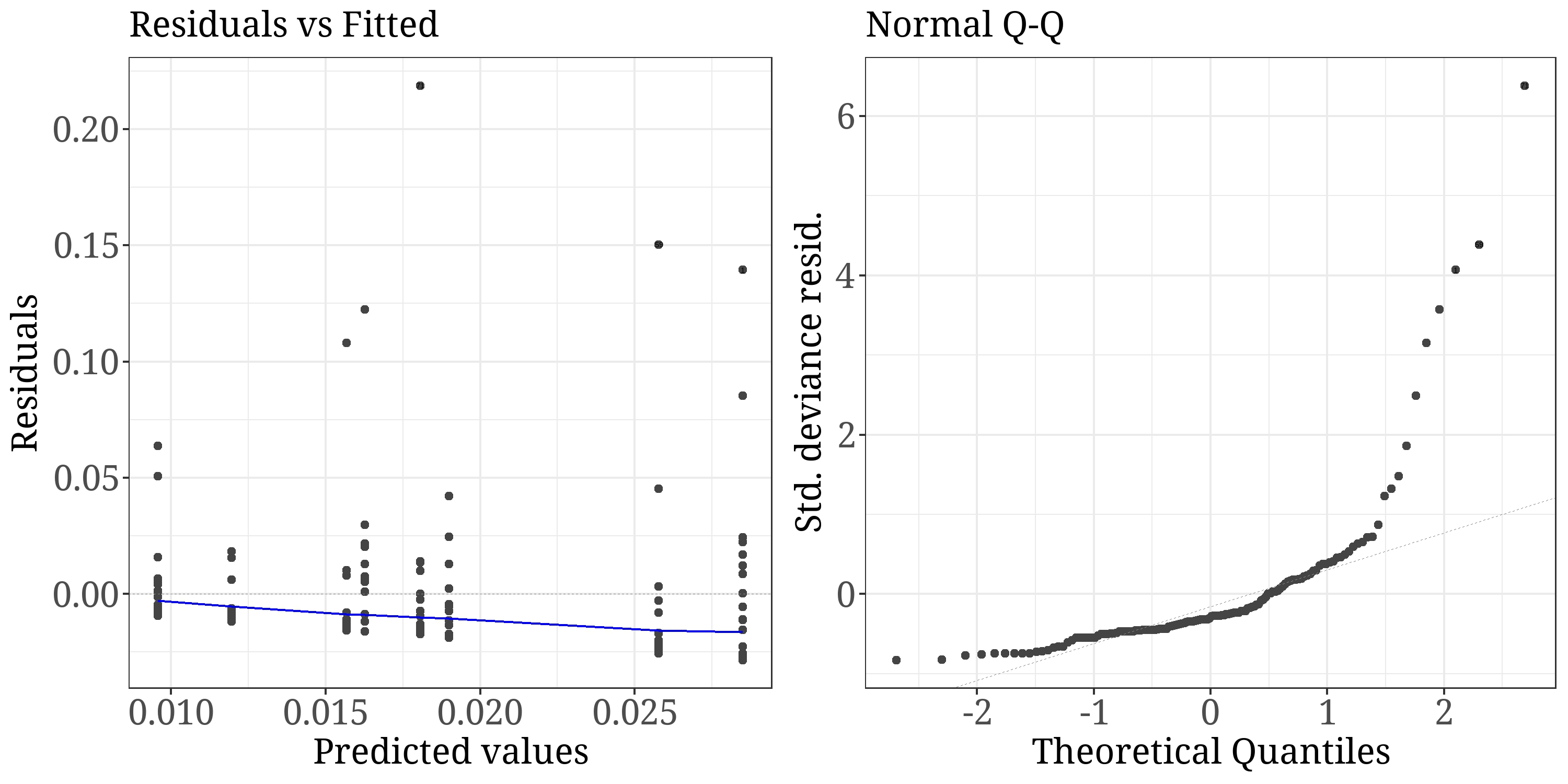
A. Original data

B. Log-transformed data


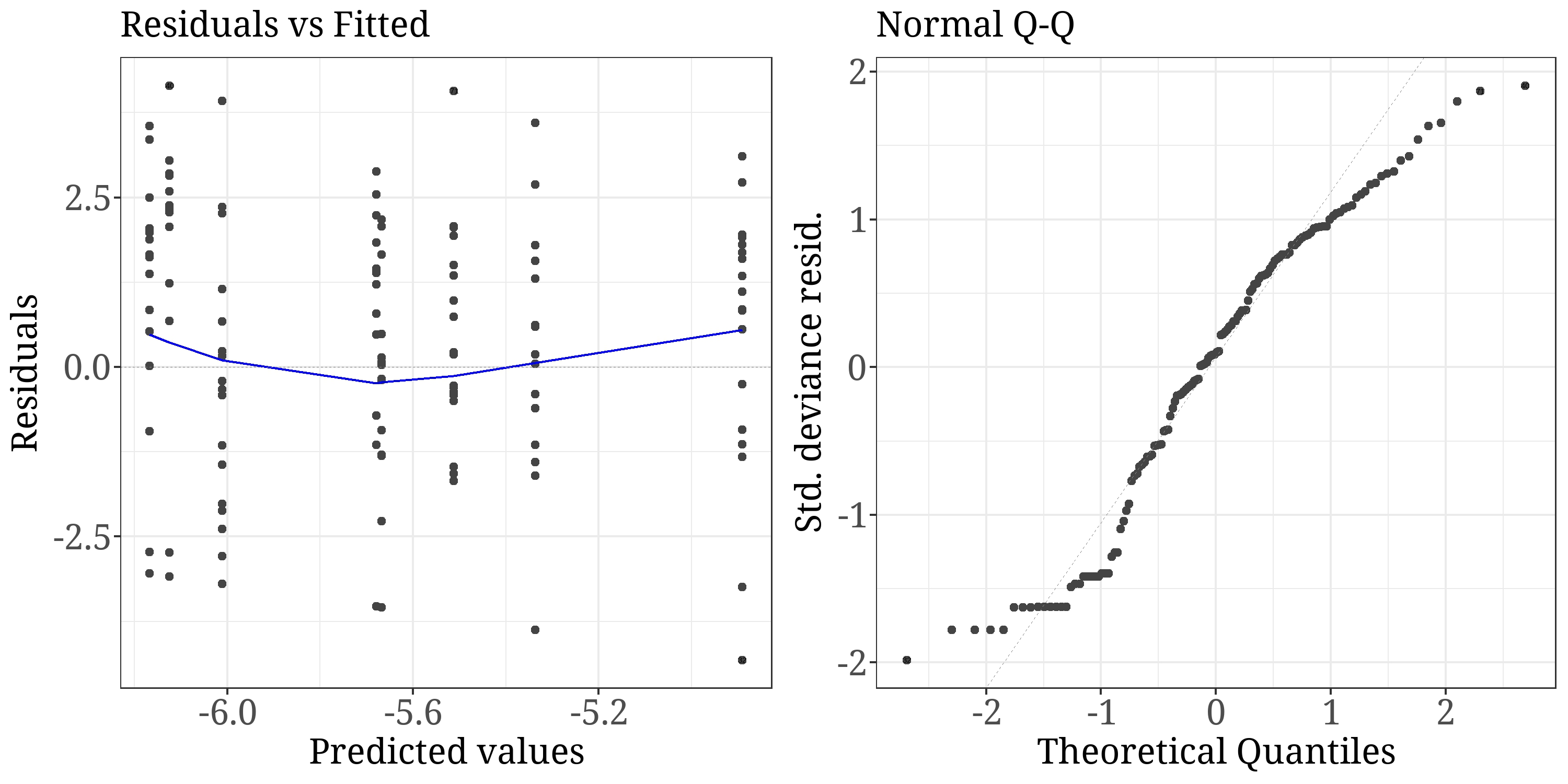


**Appendix S5.** Rarefaction (solid lines) and extrapolation (dashed lines) curves of pollinator taxonomic richness, including Formicidae, based on the number of observations. Blue lines represent direct observation data and orange lines represent interval photography data, showing diversity patterns at different taxonomic levels. The shaded areas around each curve represent the 95% confidence intervals.


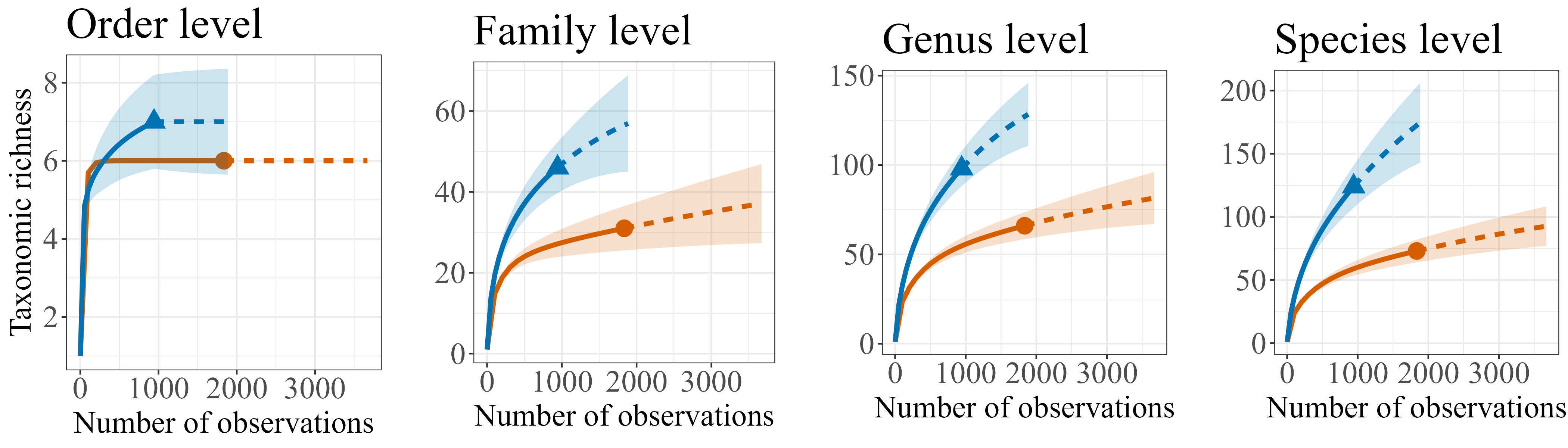


**Direct observation**

**Interval photography**


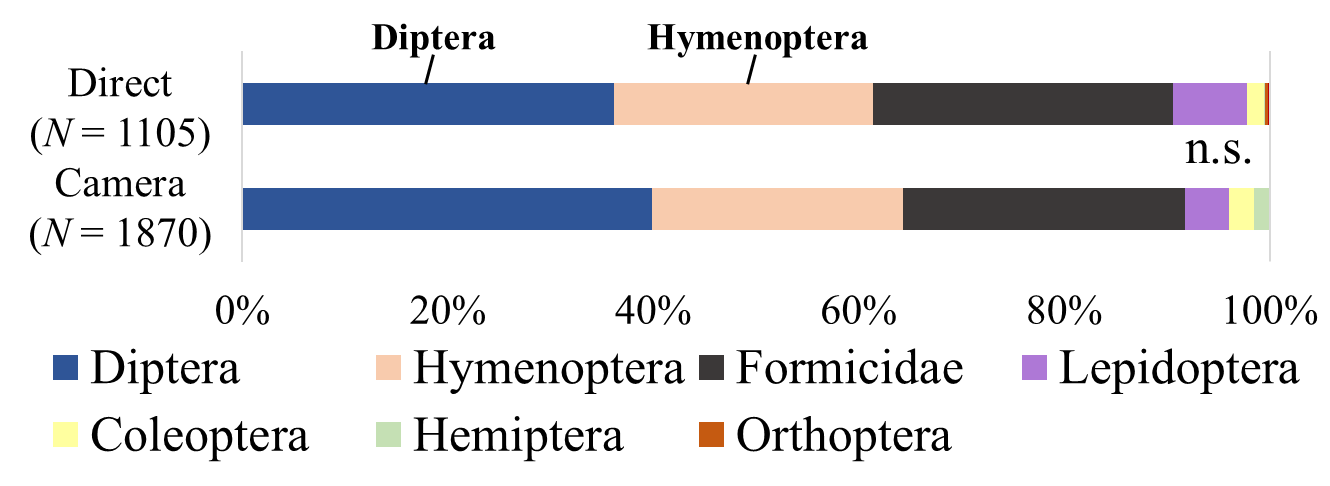
**Appendix S6.** Functional group proportions of pollinators, including Formicidae, for direct observation and interval photography in pollinator communities. The two proportions were not significantly different using Fisher’s exact probability test. Note that Hymenoptera in the figure represents Hymenoptera excluding Formicidae, which are displayed as a separate category.

**Appendix S7.** The percentage of the pollinator fauna, including Formicidae, of interval photography and direct observation. Each plant in both methods was classified according to hierarchical cluster analysis using Ward's method into Diptera-type, Hymenoptera-type, generalist-type, and ant-type based on the height of 100 in the dendrogram. Plant species that showed different pollinator types between methods are indicated in red. Note that Hymenoptera in the figure represents Hymenoptera excluding Formicidae, which are displayed as a separate category.


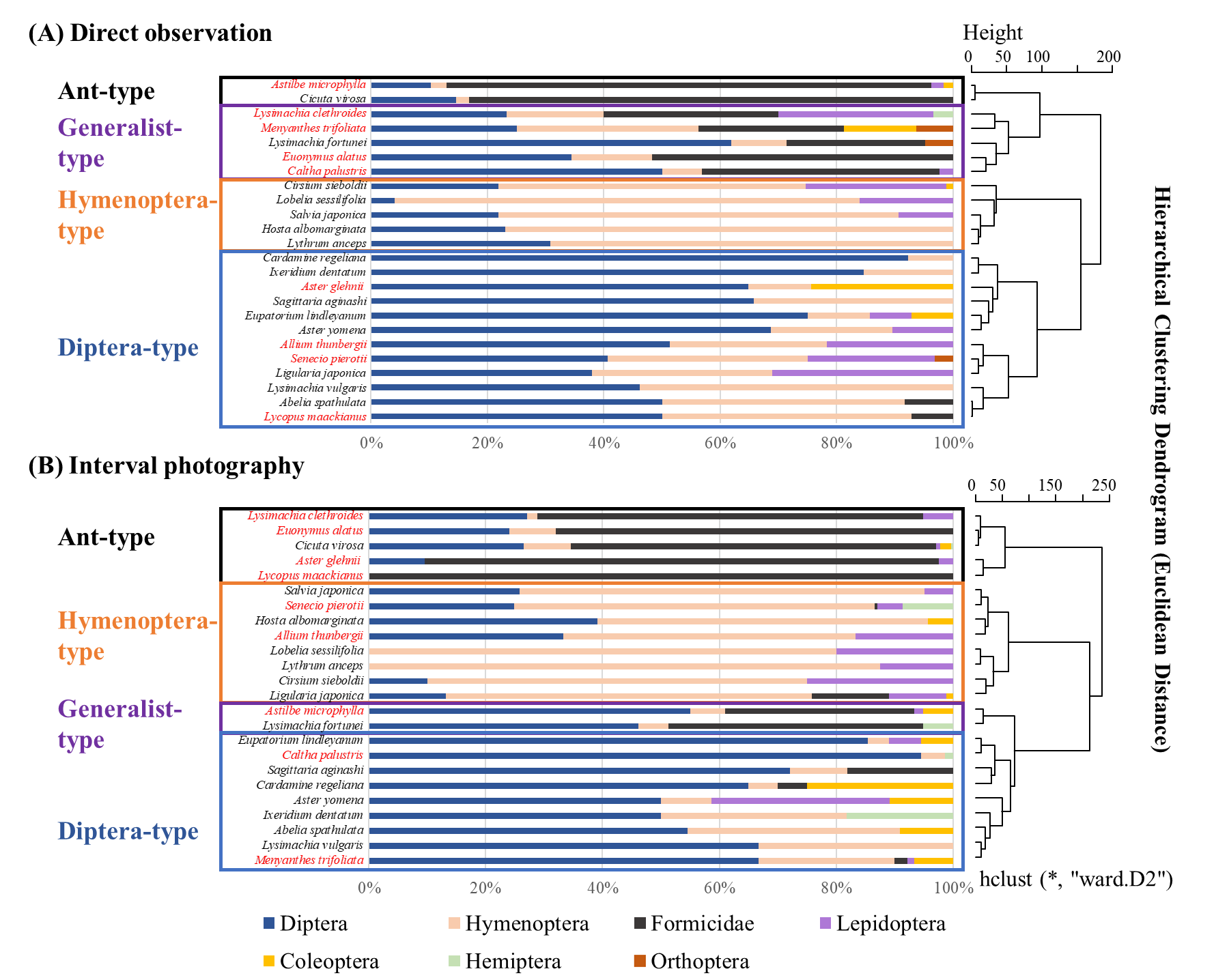


**Appendix S8.** Graph showing the relationship between the number of pollinator observations, including Formicidae, in interval photography and direct observation. Both variables were natural log-transformed using the base *e* after adding 0.0001 to each value to avoid undefined logarithms for zero values. The number of pollinator observations was expressed as observations per 15-min interval per flower. Pearson's correlation test was conducted to determine the correlation coefficient, *P*-value, and 95% confidence interval. Outliers identified using Cook's distance are highlighted in the graph.


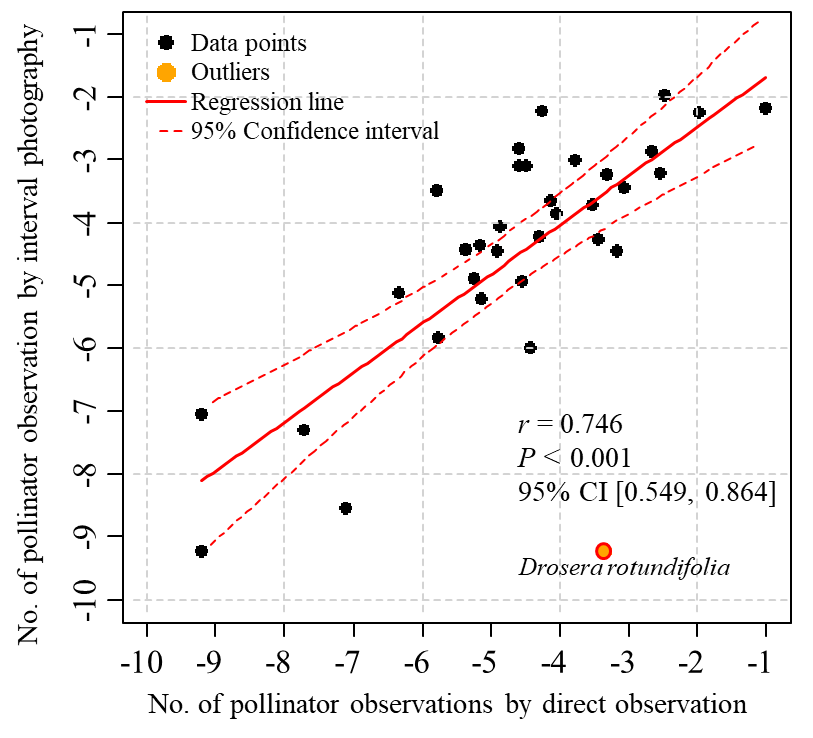


**Appendix S9.** Differences in the number of pollinator observations (including Formicidae) between direct observation and interval photography, based on generalized linear models. Pollinator counts were log-transformed after adding 1 to avoid undefined values for zeros. Two comparisons were performed: (A) between methods standardized to the same effort (15 min of direct observation vs. 15 min of interval photography), and (B) between 15 min of direct observation and 10 h of interval photography, reflecting typical operational durations in field studies. In both models, direct observation was used as the baseline level for the categorical variable method.

| Response variable | Explanatory variable | Estimate | SE | *t-*value | *P*-value |
| --- | --- | --- | --- | --- | --- |
| A: Log (no. of pollinators in direct observation and interval photography [per 15 min for both]) | Intercept | 1.30 | 0.09 | 14.34 | **<0.001** |
|  | Method (interval photography) | −1.11 | 0.13 | −8.69 | **<0.001** |
| B: Log (no. of pollinators in direct observation [per 15 min] and interval photography [per 10 h]) | Intercept | 1.30 | 0.16 | 8.03 | **<0.001** |
|  | Method (interval photography) | 0.43 | 0.23 | 1.88 | 0.06 |

**Appendix S10.** Differences in the number of pollinator observations per flower (including Formicidae) between direct observation and interval photography, based on generalized linear models. Pollinator counts were log-transformed after adding 1 to avoid undefined values for zeros. Two comparisons were performed: (A) between methods standardized to the same effort (15 min of direct observation vs. 15 min of interval photography) per flower, and (B) between 15 min of direct observation and 10 h of interval photography per flower, reflecting typical operational durations in field studies. In both models, direct observation was used as the baseline level for the categorical variable method.

| Response variable | Explanatory variable | Estimate | SE | *t-*value | *P*- value |
| --- | --- | --- | --- | --- | --- |
| A: Log (no. of pollinators in direct observation and interval photography [per 15 min per flower]) | Intercept | −4.72 | 0.34 | −13.90 | **<0.001** |
|  | Method (interval photography) | 0.14 | 0.48 | 0.28 | 0.78 |
| B: Log (no. of pollinators in direct observation [per 15 min per flower] and interval photography [per 10 h per flower]) | Intercept | −4.72 | 0.42 | −11.22 | **<0.001** |
|  | Method (interval photography) | 3.48 | 0.59 | 5.84 | **<0.001** |

**Appendix S11.** Classifiable proportions of pollinators in direct and interval photography. Numbers in parentheses indicate the number of pollinator observations for each taxon. (A) represents data excluding Formicidae, (B) represents data including Formicidae.

1. Excluding Formicidae


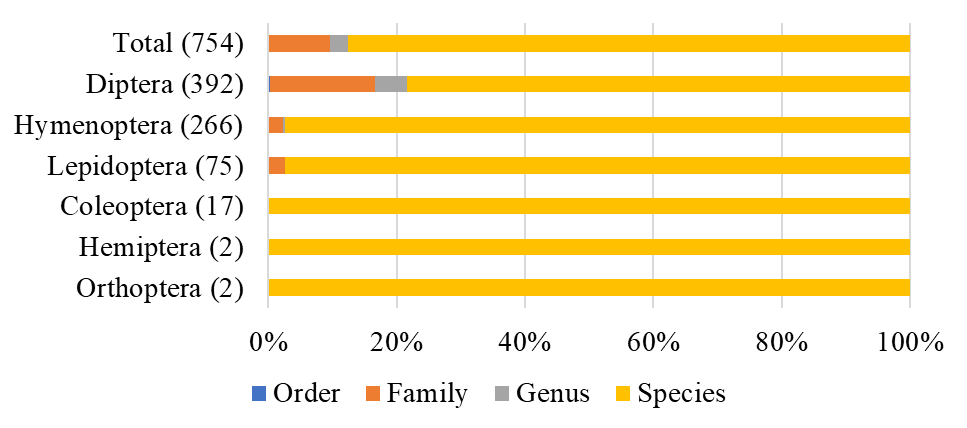
Direct observation excluding Formicidae


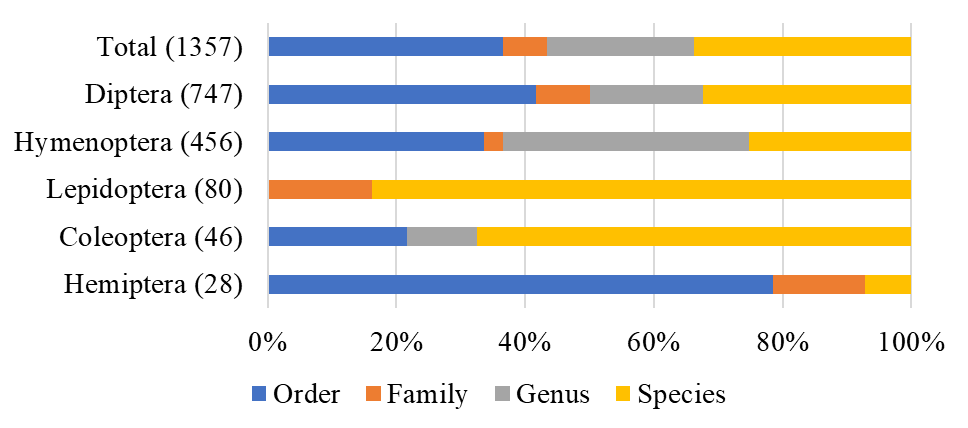
Interval photography excluding Formicidae

1. Including Formicidae


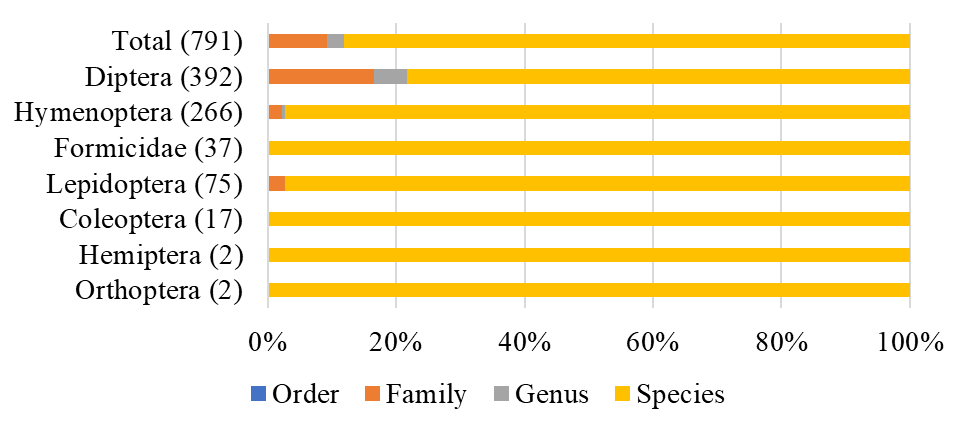
Direct observation including Formicidae

Interval photography including Formicidae


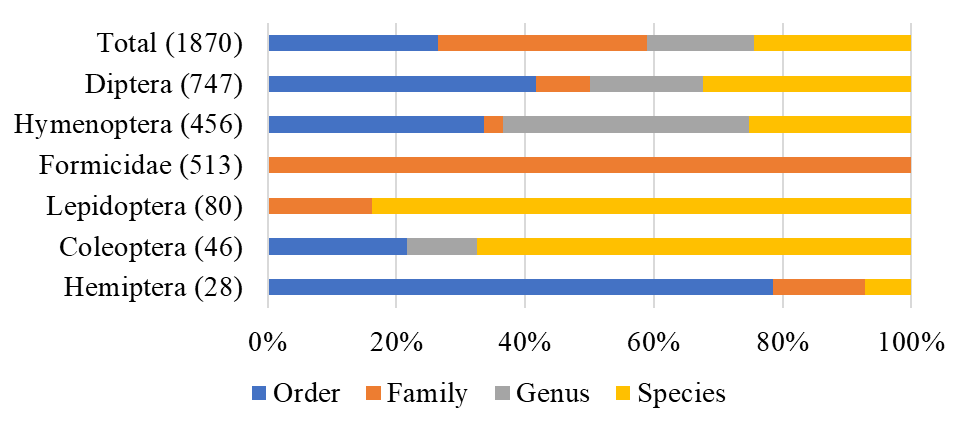


**Appendix S12.** The number of pollinator observations for each taxon in captured pollinators during direct observation and photographed pollinators using interval photography.

| Order | Family | Genus | Scientific name | Direct | Camera |
| --- | --- | --- | --- | --- | --- |
| Coleoptera | Attelabidae | *Compsapoderus* | *Compsapoderus erythrogaster* Legalov, 2003 | 1 | 1 |
| Coleoptera | Cerambycidae | *Anoploderomorpha* |  | 0 | 5 |
| Coleoptera | Cerambycidae | *Aredolpona* | *Aredolpona succedanea* N.Ohbayashi, 2007 | 0 | 3 |
| Coleoptera | Cerambycidae | *Leptura* | *Gaurotes doris* Bates, 1884 | 0 | 1 |
| Coleoptera | Cerambycidae | *Leptura* | *Leptura ochraceofasciata* (Motschulsky, 1861) | 0 | 8 |
| Coleoptera | Chrysomelidae | *Aulacophora* | *Aulacophora nigripennis* Motschulsky, 1857 | 4 | 0 |
| Coleoptera | Chrysomelidae | *Calomicrus* | *Calomicrus cyaneus* (Jacoby) | 1 | 0 |
| Coleoptera | Chrysomelidae | *Donacia* | *Donacia flemola* Goecke, 1944 | 1 | 0 |
| Coleoptera | Chrysomelidae | *Donacia* | *Donacia gracilipes* (Jacoby, 1885) | 1 | 0 |
| Coleoptera | Chrysomelidae | *Nonarthra* | *Nonarthra tibialis* (Jacoby, 1885) | 1 | 0 |
| Coleoptera | Mordellidae | *Hoshihananomia* | *Hoshihananomia perlata* (Sulzer, 1776) | 0 | 1 |
| Coleoptera | Scarabaeidae | *Cetonia* | *Cetonia roelofsi* Harold, 1880 | 1 | 2 |
| Coleoptera | Scarabaeidae | *Gametis* | *Gametis jucunda* (Faldermann, 1835) | 7 | 7 |
| Coleoptera | Scarabaeidae | *Lasiotrichius* | *Lasiotrichius succinctus* (Pallas, 1781) | 0 | 8 |
| Coleoptera |  |  |  | 0 | 10 |
| Diptera | Anthomyiidae |  |  | 2 | 0 |
| Diptera | Asilidae |  |  | 1 | 0 |
| Diptera | Bibionidae |  |  | 1 | 0 |
| Diptera | Bombyliidae | *Anthrax* | *Anthrax aygulus* Fabricius, 1805 | 1 | 0 |
| Diptera | Bombyliidae | *Bombylius* | *Bombylius (Bombylius) major* Linnaeus, 1758 | 1 | 0 |
| Diptera | Bombyliidae | *Ligyra* | *Ligyra similis* (Coquillett, 1898) | 1 | 0 |
| Diptera | Bombyliidae | *Systropus* | *Systropus nitobei* Matsumura, 1916 | 4 | 0 |
| Diptera | Bombyliidae | *Systropus* | *Systropus suzukii* Matsumura, 1916 | 1 | 0 |
| Diptera | Bombyliidae | *Systropus* |  | 0 | 33 |
| Diptera | Calliphoridae | *Isomyia* | *Isomyia senomera* (Séguy, 1949) | 5 | 4 |
| Diptera | Calliphoridae | *Lucilia* |  | 4 | 0 |
| Diptera | Calliphoridae | *Stomorhina* | *Stomorhina obsoleta* (Wiedemann, 1830) | 35 | 72 |
| Diptera | Calliphoridae |  |  | 0 | 5 |
| Diptera | Empididae |  |  | 1 | 0 |
| Diptera | Milichiidae |  |  | 6 | 0 |
| Diptera | Muscidae |  |  | 32 | 0 |
| Diptera | Mycetophilidae |  |  | 4 | 0 |
| Diptera | Psychodidae |  |  | 0 | 1 |
| Diptera | Rhagionidae |  |  | 1 | 0 |
| Diptera | Sarcophagidae |  |  | 5 | 3 |
| Diptera | Sciaridae |  |  | 1 | 0 |
| Diptera | Syrphidae | *Asarcina* | *Asarkina porcina* (Coquillett, 1898) | 1 | 5 |
| Diptera | Syrphidae | *Baccha* | *Baccha maculata* Walker, 1852 | 0 | 1 |
| Diptera | Syrphidae | *Betasyrphus* | *Betasyrphus serarius*(Wiedemann, 1830) | 3 | 0 |
| Diptera | Syrphidae | *Cheilosia* |  | 8 | 0 |
| Diptera | Syrphidae | *Didea* | *Didea alneti* (Fallén, 1817) | 0 | 5 |
| Diptera | Syrphidae | *Episyrphus* | *Episyrphus balteatus* (De Geer, 1776) | 84 | 43 |
| Diptera | Syrphidae | *Eristalis* | *Eristalis cerealis*Fabricius, 1805 | 4 | 0 |
| Diptera | Syrphidae | *Eristalis* | *Eristalis kyokoae*(Kimura, 1986) | 5 | 0 |
| Diptera | Syrphidae | *Eristalis* | *Eristalis tenax*(Linnaeus, 1758) | 1 | 0 |
| Diptera | Syrphidae | *Eristalis* |  | 0 | 1 |
| Diptera | Syrphidae | *Eumerus* |  | 6 | 0 |
| Diptera | Syrphidae | *Ferdinandea* | *Ferdinandea nigrifrons* (Egger, 1860) | 3 | 0 |
| Diptera | Syrphidae | *Mallota* | *Mallota eristaliformis*Sack, 1910 | 1 | 0 |
| Diptera | Syrphidae | *Mallota* | *Mallota takasagensis*Matsumura, 1916 | 1 | 0 |
| Diptera | Syrphidae | *Melanostoma* | *Melanostoma scalare* (Fabricius, 1794) | 3 | 0 |
| Diptera | Syrphidae | *Melanostoma* |  | 2 | 0 |
| Diptera | Syrphidae | *Mesembrius* | *Mesembrius peregrinus* (Loew, 1846) | 5 | 0 |
| Diptera | Syrphidae | *Paragus* | *Paragus haemorrhous* (Meigen, 1822) | 47 | 45 |
| Diptera | Syrphidae | *Phytomia* | *Phytomia zonata* (Fabricius, 1787) | 12 | 6 |
| Diptera | Syrphidae | *Sphaerophoria* | *Sphaerophoria indiana* Bigot, 1884 | 16 | 0 |
| Diptera | Syrphidae | *Sphaerophoria* | *Sphaerophoria macrogaster* (Thomson, 1869) | 53 | 0 |
| Diptera | Syrphidae | *Sphaerophoria* |  | 0 | 97 |
| Diptera | Syrphidae | *Spilomyia* | *Spilomyia suzukii* Matsumura, 1916 | 0 | 1 |
| Diptera | Syrphidae | *Syrphus* | *Syrphus ribesii*(Linnaeus, 1758) | 1 | 0 |
| Diptera | Syrphidae | *Syrphus* | *Syrphus torvus* Osten Sacken, 1875 | 1 | 0 |
| Diptera | Syrphidae | *Temnostoma* | *Temnostoma nitobei*Matsumura, 1916 | 1 | 0 |
| Diptera | Syrphidae | *Volucella* | *Volucella jeddona* Bigot, 1876 | 0 | 1 |
| Diptera | Syrphidae |  |  | 0 | 54 |
| Diptera | Tachinidae | *Gonia* | *Gonia chinensis*Wiedemann, 1824 | 1 | 0 |
| Diptera | Tachinidae | *Gymnocheta* | *Gymnocheta viridis*(Fallén, 1810) | 1 | 0 |
| Diptera | Tachinidae | *Gymnosoma* | *Gymnosoma rotundatum* (Linnaeus, 1758) | 1 | 27 |
| Diptera | Tachinidae | *Prosena* | *Prosena siberita*(Fabricius, 1775) | 5 | 0 |
| Diptera | Tachinidae | *Siphona* | *Siphona paludosa*Mesnil, 1960 | 7 | 0 |
| Diptera | Tachinidae | *Tachina* | *Tachina (Servillia) jakovlevi* (Portschinsky, 1882) | 1 | 4 |
| Diptera | Tachinidae | *Tachina* | *Tachina nupta* (Rondani, 1859) | 1 | 28 |
| Diptera | Tachinidae |  |  | 5 | 0 |
| Diptera | Tipulidae |  |  | 5 | 0 |
| Diptera |  |  |  | 1 | 311 |
| Hemiptera | Cicadellidae | *Bothrogonia* | *Bothrogonia ferruginea* (Fabricius, 1787) | 0 | 1 |
| Hemiptera | Cicadellidae | *Kolla* | *Kolla atramentaria* (Motschulsky, 1859) | 0 | 1 |
| Hemiptera | Coreidae | *Cletus* | *Cletus schmidti* Kiritshenko, 1916 | 1 | 0 |
| Hemiptera | Rhopalidae | *Rhopalus* | *Rhopalus sapporensis*(Matsumura, 1905) | 1 | 0 |
| Hemiptera | Rhopalidae |  |  | 0 | 4 |
| Hemiptera |  |  |  | 0 | 22 |
| Hymenoptera | Andrenidae | *Andrena* | *Andrena miyamotoi* Hirashima, 1964 | 1 | 0 |
| Hymenoptera | Andrenidae | *Andrena* |  | 1 | 0 |
| Hymenoptera | Apidae | *Amegilla* | *Amegilla florea* (Smith, 1879) | 17 | 17 |
| Hymenoptera | Apidae | *Apis* | *Apis cerana* subsp. *japonica* Radoszkowski, 1887 | 0 | 4 |
| Hymenoptera | Apidae | *Apis* | *Apis mellifera* Linnaeus, 1758 | 1 | 0 |
| Hymenoptera | Apidae | *Bombus* | *Bombus ardens* Smith, 1879 | 2 | 0 |
| Hymenoptera | Apidae | *Bombus* | *Bombus diversus* Smith, 1869 | 70 | 89 |
| Hymenoptera | Apidae | *Bombus* | *Bombus hypocrita* Pérez, 1905 | 1 | 0 |
| Hymenoptera | Apidae | *Bombus* | *Bombus ignitus* Smith, 1869 | 5 | 0 |
| Hymenoptera | Apidae | *Bombus* |  | 0 | 2 |
| Hymenoptera | Apidae | *Ceratina* | *Ceratina flavipes*Smith, 1879 | 2 | 0 |
| Hymenoptera | Apidae | *Ceratina* | *Ceratina iwatai* Yasumatsu, 1936 | 6 | 0 |
| Hymenoptera | Apidae | *Ceratina* | *Ceratina japonica* Cockerell, 1911 | 31 | 0 |
| Hymenoptera | Apidae | *Ceratina* |  | 0 | 99 |
| Hymenoptera | Apidae | *Nomada* | *Nomada comparata* Cockerell, 1911 | 1 | 0 |
| Hymenoptera | Apidae | *Nomada* | *Nomada nipponica* Yasumatsu & Hirashima, 1951 | 1 | 0 |
| Hymenoptera | Apidae | *Nomada* | *Nomada okubira* Tsuneki, 1973 | 1 | 0 |
| Hymenoptera | Apidae | *Nomada* | *Nomada taicho* Tsuneki, 1973 | 1 | 0 |
| Hymenoptera | Apidae | *Nomada* |  | 0 | 73 |
| Hymenoptera | Apidae | *Thyreus* | *Thyreus decorus* (Smith, 1852) | 0 | 3 |
| Hymenoptera | Apidae | *Xylocopa* | *Xylocopa appendiculata* Smith, 1852 | 17 | 1 |
| Hymenoptera | Apidae |  |  | 0 | 1 |
| Hymenoptera | Argidae |  |  | 0 | 1 |
| Hymenoptera | Braconidae | *Urosigalphus* |  | 3 | 0 |
| Hymenoptera | Braconidae |  |  | 2 | 0 |
| Hymenoptera | Colletidae | *Hylaeus* | *Hylaeus floralis* (Smith, 1873) | 4 | 0 |
| Hymenoptera | Eulophidae |  |  | 1 | 0 |
| Hymenoptera | Eumenidae | *Stenodynerus* | *Stenodynerus chinensis* (de Saussure, 1863) | 1 | 0 |
| Hymenoptera | Formicidae | *Camponotus* | *Camponotus hemichlaena* Yasumatsu & Brown, 1951 | 1 | 0 |
| Hymenoptera | Formicidae | *Lasius* | *Lasius japonicus* Santschi, 1941 | 36 | 0 |
| Hymenoptera | Formicidae |  |  | 0 | 513 |
| Hymenoptera | Halicitidae | *Halictus* | *Halictus Latreille*, 1804 | 4 | 0 |
| Hymenoptera | Halicitidae | *Halictus* | *Halictus tsingtouensis*Strand, 1910 | 7 | 0 |
| Hymenoptera | Halicitidae | *Lasioglossum* | *Lasioglossum (Evylaeus) japonicum* (Dalla Torre, 1896) | 3 | 0 |
| Hymenoptera | Halicitidae | *Lasioglossum* | *Lasioglossum apristum* (Vachal, 1903) | 2 | 0 |
| Hymenoptera | Halicitidae | *Lasioglossum* | *Lasioglossum caliginosum*Murao, Ebmer & Tadauchi, 2006 | 1 | 0 |
| Hymenoptera | Halicitidae | *Lasioglossum* | *Lasioglossum mutilum* (Vachal, 1903) | 14 | 0 |
| Hymenoptera | Halicitidae | *Lasioglossum* | *Lasioglossum occidens* (Smith, 1873) | 7 | 0 |
| Hymenoptera | Halicitidae | *Lasioglossum* | *Lasioglossum pallilomum* (Strand, 1914) | 13 | 0 |
| Hymenoptera | Halicitidae | *Lasioglossum* | *Lasioglossum scitulum* (Smith, 1873) | 3 | 0 |
| Hymenoptera | Halicitidae | *Lasioglossum* | *Lasioglossum sphecodicolor*Sakagami & Tadauchi, 1995 | 1 | 0 |
| Hymenoptera | Ichneumonidae | *Iseropus* | *Iseropus orientalis* Uchida, 1928 | 6 | 0 |
| Hymenoptera | Megachilidae | *Coelioxys* | *Coelioxys yanonis* Matsumura, 1912 | 2 | 0 |
| Hymenoptera | Megachilidae | *Coelioxys* |  | 0 | 1 |
| Hymenoptera | Megachilidae | *Megachile* | *Megachile tsurugensis* Cockerell, 1924 | 21 | 0 |
| Hymenoptera | Melittidae | *Macropis* | *Macropis tibialis* Yasumatsu & Hirashima, 1956 | 6 | 0 |
| Hymenoptera | Melittidae | *Melitta* | *Melitta ezoana*Yasumatsu & Hirashima, 1956 | 3 | 0 |
| Hymenoptera | Scoliidae | *Megacampsomeris* | *Megacampsomeris grossa*(Fabricius, 1804) | 3 | 0 |
| Hymenoptera | Scoliidae |  |  | 0 | 12 |
| Hymenoptera | Vespidae | *Eumenes* | *Eumenes micado* Cameron, 1904 | 1 | 0 |
| Hymenoptera |  |  |  | 0 | 153 |
| Lepidoptera | Callidulidae | *Pterodecta* | *Pterodecta felderi* (Bremer, 1864) | 1 | 0 |
| Lepidoptera | Crambidae | *Bocchoris* | *Bocchoris inspersalis* Zeller, 1852 | 0 | 8 |
| Lepidoptera | Crambidae | *Spoladea* | *Spoladea recurvalis* (Fabricius, 1775) | 0 | 3 |
| Lepidoptera | Epicopeiidae | *Psychostrophia* | *Psychostrophia melanargia* Butler, 1877 | 0 | 7 |
| Lepidoptera | Hesperiidae | *Ochlodes* | *Ochlodes ochracea* (Bremer, 1861) | 3 | 3 |
| Lepidoptera | Hesperiidae | *Parnara* | *Parnara guttatus* (Bremer & Grey, 1853) | 1 | 1 |
| Lepidoptera | Hesperiidae | *Pelopidas* | *Pelopidas mathias* (Fabricius, 1798) | 3 | 3 |
| Lepidoptera | Hesperiidae | *Polytremis* | *Polytremis pellucida* (Murray, 1875) | 7 | 6 |
| Lepidoptera | Hesperiidae | *Thymelicus* | *Thymelicus leonina* (Butler, 1878) | 1 | 0 |
| Lepidoptera | Hesperiidae |  |  | 0 | 8 |
| Lepidoptera | Lycaenidae | *Everes* | *Everes argiades* (Pallas, 1771) | 1 | 0 |
| Lepidoptera | Lycaenidae | *Lycaena* | *Lycaena phlaeas* (Linnaeus, 1761) | 8 | 10 |
| Lepidoptera | Nymphalidae | *Argyreus* | *Argyreus hyperbius* (Linnaeus, 1763) | 1 | 0 |
| Lepidoptera | Nymphalidae | *Argyronome* | *Argyronome ruslana* (Motschulsky, 1866) | 17 | 0 |
| Lepidoptera | Nymphalidae | *Fabriciana* | *Fabriciana adippe* (Denis & Schiffermüller) 1775 | 4 | 5 |
| Lepidoptera | Nymphalidae | *Mycalesis* | *Mycalesis gotama*Moore, 1857 | 1 | 0 |
| Lepidoptera | Nymphalidae | *Nephargynnis* | *Nephargynnis anadyomene* (C. Felder et R. Felder, 1862) | 1 | 0 |
| Lepidoptera | Nymphalidae | *Neptis* | *Neptis sappho* Pallas, 1771 | 1 | 0 |
| Lepidoptera | Nymphalidae | *Polygonia* | *Polygonia c-aureum* (Linnaeus, 1758) | 1 | 1 |
| Lepidoptera | Nymphalidae | *Vanessa* | *Vanessa indica* (Herbst, 1794) | 2 | 0 |
| Lepidoptera | Nymphalidae | *Ypthima* | *Ypthima argus* Butler, 1878 | 1 | 3 |
| Lepidoptera | Papilionidae | *Papilio* | *Papilio helenus* Linnaeus, 1758 | 2 | 4 |
| Lepidoptera | Papilionidae | *Papilio* | *Papilio maackii* Ménétriés, 1859 | 0 | 1 |
| Lepidoptera | Papilionidae | *Papilio* | *Papilio machaon* Linnaeus, 1758 | 1 | 0 |
| Lepidoptera | Papilionidae | *Papilio* | *Papilio protenor* Cramer, 1775 | 0 | 2 |
| Lepidoptera | Papilionidae |  |  | 0 | 1 |
| Lepidoptera | Pieridae | *Eurema* | *Eurema mandarina* (de l'Orza, 1869) | 8 | 1 |
| Lepidoptera | Pieridae | *Pieris* | *Pieris melete* Ménétriès, 1857 | 4 | 1 |
| Lepidoptera | Pieridae | *Pieris* | *Pieris rapae* (Linnaeus, 1758) | 1 | 1 |
| Lepidoptera | Pieridae |  |  | 0 | 2 |
| Lepidoptera | Pterophoridae |  |  | 2 | 1 |
| Lepidoptera | Sphingidae | *Macroglossum* | *Macroglossum bombylans* Boisduval | 0 | 1 |
| Lepidoptera | Sphingidae | *Macroglossum* | *Macroglossum pyrrhosticta* Butler, 1875 | 1 | 6 |
| Lepidoptera | Sphingidae | *Macroglossum* | *Macroglossum saga* Butler, 1878 | 1 | 0 |
| Lepidoptera | Sphingidae | *Neogurelca* | *Neogurelca himachala* (Butler, 1875) | 1 | 0 |
| Lepidoptera | Sphingidae |  |  | 0 | 1 |
| Orthoptera | Tettigoniidae | *Eobiana* | *Eobiana engelhardti*subsp. *subtropica* (Bey-Bienko, 1949) | 1 | 0 |
| Orthoptera | Tettigoniidae | *Gampsocleis* | *Gampsocleis buergeri*(Haan, 1843) | 1 | 0 |
|  |  |  | Total | 791 | 1870 |

**Appendix S13.** The number of pollinator species in each order identified by direct observation and interval photography.

| Order | Direct | Camera | Total |
| --- | --- | --- | --- |
| Diptera | 32 | 13 | 36 |
| Hymenoptera | 34 | 5 | 36 |
| Formicidae | 2 | 0 | 2 |
| Lepidoptera | 25 | 24 | 31 |
| Coleoptera | 8 | 10 | 13 |
| Hemiptera | 2 | 2 | 4 |
| Orthoptera | 2 | 0 | 2 |
| Total | 105 | 54 | 124 |

**Appendix S14.** Comparison of sampling coverage and taxonomic richness between direct observation and interval photography methods across taxonomic hierarchies. The number of plant species, number of pollinator taxa, sampling coverage, and taxonomic richness values for direct observation and interval photography are shown at (a) order level, (b) family level, (c) genus level, and (d) species level. I/D indicates the value obtained by dividing taxonomic richness of interval photography by taxonomic richness of direct observation. (A) represents data excluding Formicidae, (B) represents data including Formicidae.

(A) Excluding Formicidae

| (a) Order level |  |  |  |
| --- | --- | --- | --- |
|  | Direct observation | Interval photography | I/D |
| No. of plant species | 22 | 22 | - |
| No. of pollinator taxa | 6 | 5 | - |
| Sampling coverage | 1 | 1 | - |
| Taxonomic richness | 6 | 5 | 0.83 |
|  |  |  |  |
| (b) Family level |  |  |  |
|  | Direct observation | Interval photography | I/D |
| No. of plant species | 22 | 22 | - |
| No. of pollinator taxa | 45 | 30 | - |
| Sampling coverage | 0.97 | 1.00 | - |
| Taxonomic richness | 81.06 | 54.48 | 0.67 |
|  |  |  |  |
| (c) Genus level |  |  |  |
|  | Direct observation | Interval photography | I/D |
| No. of plant species | 22 | 22 | - |
| No. of pollinator taxa | 90 | 64 | - |
| Sampling coverage | 0.93 | 0.99 | - |
| Taxonomic richness | 165.92 | 104.47 | 0.63 |
|  |  |  |  |
| (d) Species level |  |  |  |
|  | Direct observation | Interval photography | I/D |
| No. of plant species | 22 | 22 | - |
| No. of pollinator taxa | 115 | 71 | - |
| Sampling coverage | 0.90 | 0.98 | - |
| Taxonomic richness | 294.69 | 123.86 | 0.42 |

(B) Including Formicidae

| (a) Order level |  |  |  |
| --- | --- | --- | --- |
|  | Direct observation | Interval photography | I/D |
| No. of plant species | 24 | 24 | - |
| No. of pollinator taxa | 7 | 6 | - |
| Sampling coverage | 1 | 1 | - |
| Taxonomic richness | 7 | 6 | 0.86 |
|  |  |  |  |
| (b) Family level |  |  |  |
|  | Direct observation | Interval photography | I/D |
| No. of plant species | 24 | 24 | - |
| No. of pollinator taxa | 46 | 31 | - |
| Sampling coverage | 0.98 | 1.00 | - |
| Taxonomic richness | 68.48 | 55.49 | 0.81 |
|  |  |  |  |
| (c) Genus level |  |  |  |
|  | Direct observation | Interval photography | I/D |
| No. of plant species | 24 | 24 | - |
| No. of pollinator taxa | 98 | 66 | - |
| Sampling coverage | 0.96 | 0.99 | - |
| Taxonomic richness | 162.59 | 111.1 | 0.68 |
|  |  |  |  |
| (d) Species level |  |  |  |
|  | Direct observation | Interval photography | I/D |
| No. of plant species | 24 | 24 | - |
| No. of pollinator taxa | 124 | 73 | - |
| Sampling coverage | 0.93 | 0.99 | - |
| Taxonomic richness | 271.69 | 130.57 | 0.48 |

**Appendix S15.** Observation-based rarefaction (solid lines) and extrapolation (dashed lines) curves showing pollinator diversity at different taxonomic levels for each plant species. Blue lines represent direct observation data and orange lines represent interval photography data, showing diversity patterns at different taxonomic levels. The shaded areas around each curve represent the 95% confidence intervals. (A) represents data excluding Formicidae, (B) represents data including Formicidae.

(A) Excluding Formicidae


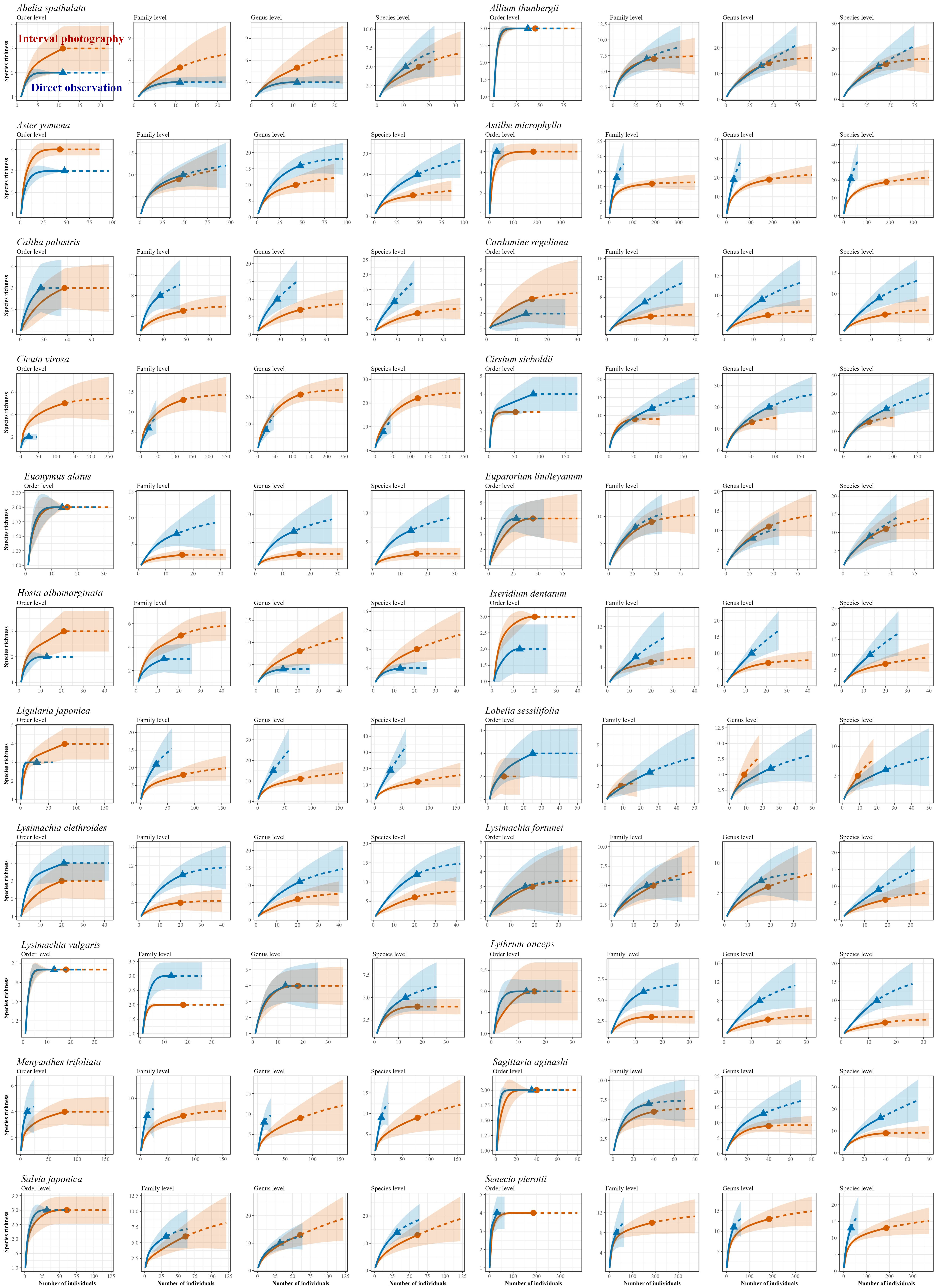


(B) Including Formicidae


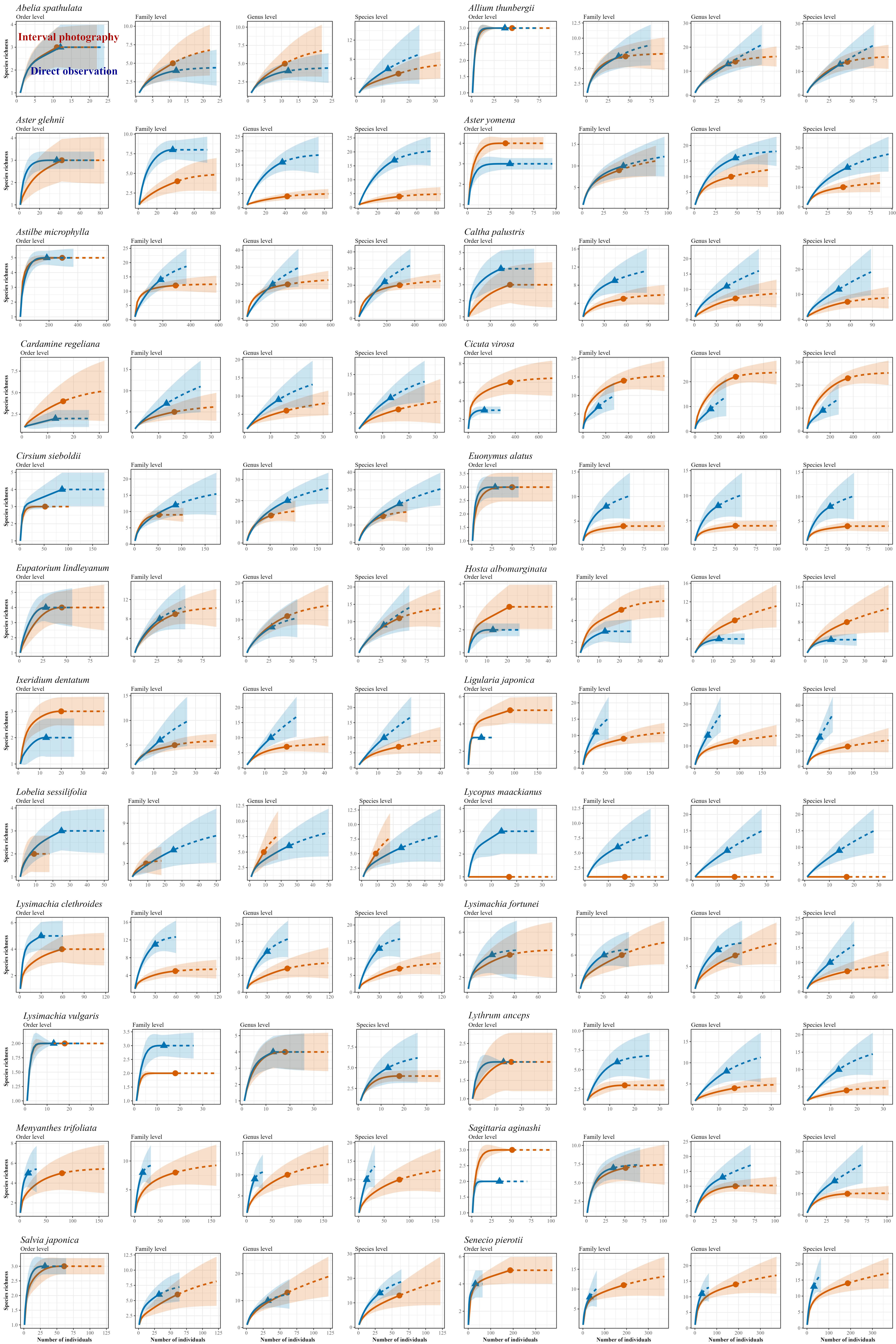


**Appendix S16.** Comparison of sampling coverage and taxonomic richness between direct observation and interval photography methods for each plant species across taxonomic hierarchies. Number of plant species, number of pollinator taxa, sampling coverage, and taxonomic richness values for direct observation and interval photography are shown at (a) order level, (b) family level, (c) genus level, and (d) species level. Values are presented as mean ± standard error. Differences between the two methods were tested using paired *t*-test. (A) represents data excluding Formicidae, (B) represents data including Formicidae.

(A) Excluding Formicidae

| (a) Order level |  |  |  |  |
| --- | --- | --- | --- | --- |
|  | Direct observation | Interval photography | *t*-value | *P*-value |
| No. of plant species | 22 | 22 | - | - |
| No. of pollinator taxa | 6 | 5 | - | - |
| Sampling coverage | 0.999 ± 0.001 | 1.000 ± 0.000 | - | - |
| Taxonomic richness | 2.90 ± 0.19 | 3.14 ± 0.18 | −1.23 | 0.23 |
|  |  |  |  |  |
| (b) Family level |  |  |  |  |
|  | Direct observation | Interval photography | *t*-value | *P*-value |
| No. of plant species | 22 | 22 | - | - |
| No. of pollinator taxa | 45 | 30 | - | - |
| Sampling coverage | 0.94 ± 0.01 | 0.99 ± 0.00 | - | - |
| Taxonomic richness | 9.19 ± 0.82 | 7.21 ± 0.72 | 2.54 | **< 0.05** |
|  |  |  |  |  |
| (c) Genus level |  |  |  |  |
|  | Direct observation | Interval photography | *t*-value | *P*-value |
| No. of plant species | 22 | 22 | - | - |
| No. of pollinator taxa | 90 | 64 | - | - |
| Sampling coverage | 0.89 ± 0.03 | 0.97 ± 0.01 | - | - |
| Taxonomic richness | 13.71 ± 1.48 | 11.12 ± 1.22 | 1.94 | 0.07 |
|  |  |  |  |  |
| (d) Species level |  |  |  |  |
|  | Direct observation | Interval photography | *t*-value | *P*-value |
| No. of plant species | 22 | 22 | - | - |
| No. of pollinator taxa | 115 | 71 | - | - |
| Sampling coverage | 0.83 ± 0.03 | 0.97 ± 0.01 | - | - |
| Taxonomic richness | 16.76 ± 1.76 | 11.33 ± 1.26 | 3.62 | **< 0.01** |

(B) Including Formicidae

| (a) Order level |  |  |  |  |
| --- | --- | --- | --- | --- |
|  | Direct observation | Interval photography | *t*-value | *P*-value |
| No. of plant species | 24 | 24 | - | - |
| No. of pollinator taxa | 7 | 6 | - | - |
| Sampling coverage | 0.999 ± 0.000 | 0.999 ± 0.001 | - | - |
| Taxonomic richness | 3.24 ± 0.21 | 3.51 ± 0.25 | −1.11 | 0.28 |
|  |  |  |  |  |
| (b) Family level |  |  |  |  |
|  | Direct observation | Interval photography | *t*-value | *P*-value |
| No. of plant species | 24 | 24 | - | - |
| No. of pollinator taxa | 46 | 31 | - | - |
| Sampling coverage | 0.95 ± 0.01 | 0.99 ± 0.00 | - | - |
| Taxonomic richness | 9.45 ± 0.01 | 7.36 ± 0.76 | 2.85 | **< 0.01** |
|  |  |  |  |  |
| (c) Genus level |  |  |  |  |
|  | Direct observation | Interval photography | *t*-value | *P*-value |
| No. of plant species | 24 | 24 | - | - |
| No. of pollinator taxa | 98 | 66 | - | - |
| Sampling coverage | 0.90 ± 0.03 | 0.97 ± 0.01 | - | - |
| Taxonomic richness | 14.33 ± 1.36 | 10.93 ± 1.25 | 2.49 | **< 0.05** |
|  |  |  |  |  |
| (d) Species level |  |  |  |  |
|  | Direct observation | Interval photography | *t*-value | *P*-value |
| No. of plant species | 24 | 24 | - | - |
| No. of pollinator taxa | 124 | 73 | - | - |
| Sampling coverage | 0.85 ± 0.03 | 0.97 ± 0.01 | - | - |
| Taxonomic richness | 17.22 ± 1.59 | 11.12 ± 1.28 | 4.21 | **< 0.001** |

**Appendix S17.** Plant species with different entomophilic types as recorded using direct observation and interval photography, and statistical significance in the proportion of pollinator groups based on Fisher’s exact test with Bonferroni correction. ^n.s.^ not significant, ^*^ *P* < 0.05, ^**^ *P* < 0.01, ^***^ *P* < 0.001. (A) represents data excluding Formicidae, (B) represents data including Formicidae.

1. Excluding Formicidae

| Flower species | Direct observation | Interval photography | *P*-value of Fisher’s exact test | Significance after Bonferroni correction |
| --- | --- | --- | --- | --- |
| *Allium thunbergii* | G-type | H-type | 0.10 | n.s. |
| *Aster yomena* | D-type | G-type | 0.002 | * |
| *Cicuta virosa* | D-type | G-type | 0.23 | n.s. |
| *Euonymus alatus* | D-type | G-type | 1.00 | n.s. |
| *Ixeridium dentatum* | D-type | G-type | 0.12 | n.s. |
| *Ligularia japonica* | G-type | H-type | 0.0004 | ** |
| *Lysimachia clethroides* | G-type | D-type | 0.01 | n.s. |
| *Senecio pierotii* | G-type | H-type | 0.02 | n.s. |

1. Including Formicidae

| Flower species | Direct observation | Interval photography | *P*-value of Fisher’s exact test | Significance after Bonferroni correction |
| --- | --- | --- | --- | --- |
| *Allium thunbergii* | D-type | H-type | 0.10 | n.s. |
| *Aster glehnii* | D-type | A-type | 0.000000003 | *** |
| *Astilbe microphylla* | A-type | G-type | 0.0000000000000002 | *** |
| *Caltha palustris* | G-type | D-type | 0.000000003 | *** |
| *Euonymus alatus* | G-type | A-type | 0.36 | n.s. |
| *Lycopus maackianus* | D-type | A-type | 0.00000007 | *** |
| *Lysimachia clethroides* | G-type | A-type | 0.04 | n.s. |
| *Menyanthes trifoliata* | G-type | D-type | 0.001 | * |
| *Senecio pierotii* | D-type | H-type | 0.01 | n.s. |

**Appendix S18.** Differences in the number of pollinator observations per flower (excluding Formicidae) between direct observation and interval photography, based on generalized linear models. Pollinator counts were log-transformed after adding 1 to avoid undefined values for zeros. Two comparisons were performed: (A) between methods standardized to the same effort (15 min of direct observation vs. 15 min of interval photography) per flower, and (B) between 15 min of direct observation and 10 h of interval photography per flower, reflecting typical operational durations in field studies. In both models, direct observation was used as the baseline level for the categorical variable method.

| Response variable | Explanatory variable | Estimate | SE | *t*-value | *P*-value |
| --- | --- | --- | --- | --- | --- |
| A: Log (no. of pollinators in direct observation and interval photography [per 15 min per flower]) | Intercept | −4.88 | 0.35 | −13.87 | **<0.001** |
|  | Method (interval photography) | −0.02 | 0.50 | −0.05 | 0.96 |
| B: Log (no. of pollinators in direct observation [per 15 min per flower] and interval photography [per 10 h per flower]) | Intercept | −4.88 | 0.45 | −10.92 | **<0.001** |
|  | Method (interval photography) | 3.21 | 0.63 | 5.07 | **<0.001** |
